# New Biological Insights on X and Y Chromosome-Bearing Spermatozoa

**DOI:** 10.3389/fcell.2019.00388

**Published:** 2020-01-21

**Authors:** Md Saidur Rahman, Myung-Geol Pang

**Affiliations:** Department of Animal Science and Technology and BET Research Institute, Chung-Ang University, Anseong, South Korea

**Keywords:** X spermatozoa, Y spermatozoa, sperm function, proteome, genome

## Abstract

A spermatozoon is a male germ cell capable of fertilizing an oocyte and carries genetic information for determining the sex of the offspring. It comprises autosomes and an X (X spermatozoa) or a Y chromosome (Y spermatozoa). The origin and maturation of both X and Y spermatozoa are the same, however, certain differences may exist. Previous studies proposed a substantial difference between X and Y spermatozoa, however, recent studies suggest negligible or no differences between these spermatozoa with respect to ratio, shape and size, motility and swimming pattern, strength, electric charge, pH, stress response, and aneuploidy. The only difference between X and Y spermatozoa lies in their DNA content. Moreover, recent proteomic and genomic studies have identified a set of proteins and genes that are differentially expressed between X and Y spermatozoa. Therefore, the difference in DNA content might be responsible for the differential expression of certain genes and proteins between these cells. In this review, we have compiled our present knowledge to compare X and Y spermatozoa with respect to their structural, functional, and molecular features. In addition, we have highlighted several areas that could be explored in future studies in this field.

## Introduction

**FIGURE 1:**
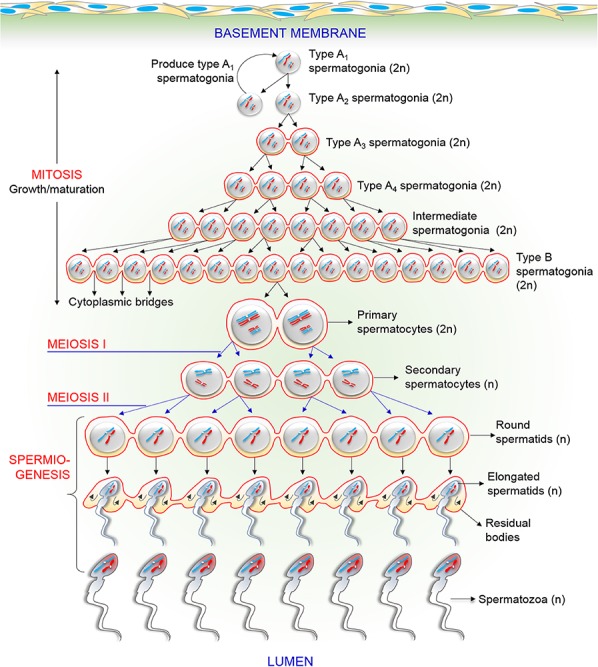
Schematic drawing of the process of various events in male germ cells during spermatogenesis and spermiogenesis. The figure particularly displays the existence of intercellular bridges among spermatogenic cells. Each generation of cells is connected by intercellular bridges, thus, it divides synchronously in cohorts. See the main text for a detailed description.

A spermatozoon is a male reproductive cell that is produced in testis by highly orchestrated processes called spermatogenesis and spermiogenesis. During spermatogenesis, undifferentiated spermatogonia (stem cells) transform into type A_l_ spermatogonia (differentiated cells). Eventually, by the process of several mitotic cell divisions, type A_l_ spermatogonia become type B spermatogonia (Leblond and Clermont, 1952; Oakberg, 1956). Type B spermatogonia subsequently undergo a final round of mitosis to form the primary spermatocytes (only two cells are shown) that further proceed to meiosis (Figure 1). Through the first meiotic cell division, the primary spermatocyte yields two secondary spermatocytes, which then enter the second meiotic division and divides into four round spermatids that contain either the X or Y chromosomes (Leblond and Clermont, 1952). Finally, the haploid round spermatids differentiate to elongated spermatids and ultimately into spermatozoa by the process of spermiogenesis (Hendriksen, 1999). During this entire process, the spermatogenic cells migrate from the basement membrane toward the center of the seminiferous tubule and released into the lumen. It has been reported that cytokinesis is not complete during mitotic and meiotic divisions of these processes (Hendriksen, 1999). As shown in Figure 1, spermatogenic cells from the same type of A_l_ spermatogonium form a syncytium and are connected by intercellular bridges that persist until the end of spermatogenesis (Braun et al., 1989). This intercellular bridge permits free cytoplasmic communication among the cells with different genotypes. Because ions and molecules (including genes and proteins) readily pass through these intercellular bridges, each cell containing either X or Y chromosome are matured synchronously (Braun et al., 1989; Jasin and Zalamea, 1992). Therefore, the origin, maturation, and functions of both X and Y chromosome-bearing spermatozoa are mostly identical.

Subsequently mature spermatozoa are released in semen during ejaculation, and is capable of fertilizing an oocyte, followed by contributing half of the genetic material to the offspring (Clapham, 2013; Rahman et al., 2013). Based on the chromosomal content, spermatozoa are of two types, that is, those bearing the X chromosome (X spermatozoa) and Y chromosome (Y spermatozoa) (Shettles, 1960; Gellatly, 2009). If the X spermatozoon combines with the mother’s X chromosome, the resulting offspring is a baby girl (XX), whereas if Y spermatozoon fertilizes the mother’s oocyte, the resulting offspring is a baby boy (Gellatly, 2009). Certain preliminary studies reported several morphological differences between the X and Y spermatozoa using phase-contrast microscopy (Shettles, 1960; Cui and Matthews, 1993; Cui, 1997); however, most of the recent studies indicate that no major differences exist between the two sperm types (Hossain et al., 2001; You et al., 2017) except their DNA content. The discrepancy in these results, from the early and recent studies, is presumably due to relatively non-specific methods used by the early investigators to differentiate between the X and Y spermatozoa (Hossain et al., 2001). Therefore, to understand the real differences between the two sperm types, a thorough discussion of both cells needs to be emphasized. In the present review, we summarized the existing scientific evidence to compare the X and Y spermatozoa considering their morphophysiological and molecular characteristics. In addition, we have highlighted the proteomics and genomics aspects of both cells, and investigated their clinical significance, in order to predict whether this difference could explain the occurrence of particular diseases in a sex-specific-manner.

## Search Scheme and Article Selection

PubMed search engine was used to thoroughly search the MEDLINE database for literature on X and/or Y spermatozoa using the following search terms: ratio, shape, size, gender selection, motility, swimming pattern, velocity, CASA, FISH, flow cytometric analysis, Percoll gradient, albumin gradient, swim-up method, viability, electrophobicity, electronegativity, pH tolerance, surface properties, Y-specific antigen, HY antigen, stress response, oxidative stress, endocrine disruptors, pesticide exposure, environmental toxicants, heat stress, DNA damage, chromosomal abnormality, aneuploidy, XX aneuploidy, XY aneuploidy, YY aneuploidy, proteomics, disease, gender-specific disease, and genomics. Full-text articles and abstracts in English language on X and Y spermatozoa published before December, 2019, were included in the review after screening their content. All article types such as original articles, reviews, letter to the editor, editorials, opinions, and debates were included in the review. Retracted papers were excluded by thoroughly checking the corresponding journal websites.

## Morphophysiological Characteristics of X and Y Spermatozoa

A mature mammalian spermatozoon comprises three distinct parts, namely, head, mid-piece, and tail (containing genetic material, mitochondria, and axial filament, respectively). Due to their unique organization, spermatozoa are different from the other cells. In this section, we will compare X and Y spermatozoa based on their morphological and physiological characteristics.

### Ratio

During mammalian spermatogenesis, meiosis produces 50:50 ratio of X and Y spermatozoa according to Mendelian segregation. Therefore, the natural sex ratio during spermatogenesis is expected to be 1:1 (Umehara et al., 2019). An intensive literature search produced three major findings on the ratio of X and Y spermatozoa: (1) proportion of X spermatozoa was higher than that of Y spermatozoa (Martin et al., 1983; Bibbins et al., 1988), (2) proportion of Y spermatozoa was higher than that of X spermatozoa (Landrum and Shettles, 1960; Shettles, 1960; Quinlivan and Sullivan, 1974), and (3) no difference existed in the proportion of the two sperm types (Van Kooij and Van Oost, 1992; Goldman et al., 1993; Han et al., 1993a).

It has been reported that an uncharacterized (unidentified) gene controlled the ratio of X and Y spermatozoa such that men with more brothers had a higher probability of having sons and those with more sisters had a higher probability of having daughters (Gellatly, 2009); however, these findings are mostly hypothetical, and presence of such a gene has not yet been confirmed. Moreover, a non-significant increase of Y spermatozoa in men with only sons (>3) or the X spermatozoa in men with only daughters (>3) has been reported in another investigation (Irving et al., 1999). Several previous studies from 1970 to 1980 suggested that paternal age differentially affected the ratio of X and Y spermatozoa, thus altering the secondary sex ratio of the offspring in a particular population (Erickson, 1976; James and Rostron, 1985; Ruder, 1985). Nevertheless, this finding was also proven to be imprecise by other contemporaries (Curtsinger et al., 1983; Martin et al., 1995a, b). Apart from the debate, it has been reported that an active gene transcription occurs selectively in the chromosomes (including sex chromosomes) of haploid round spermatids (Hu and Namekawa, 2015). Therefore, if the composition of sex chromosomes has changed due to the post-meiotic modifications in the gene expression and differential survival of spermatozoa during epididymal maturation, these may affect the expected ratio (Bean, 1990). In a recent study, Umehara et al. (2019) reported that ligand activation of Toll-like receptors 7/8 (TLR7/8), selectively encoded by the X chromosome, significantly suppress the motility of X spermatozoa without altering their ability of fertilization. This procedure allows producing over 90% of the male embryos following *in vitro* fertilization using ligand-selected highly motile spermatozoa. In another study using knockout (KO) mice model, Rathje et al. (2019) reported that partial deletions of the Y chromosome (Yqdel) in males produce an equal number of X and Y spermatozoa. Although both sperm types are equally capable of fertilizing oocytes once at the site of fertilization, they exhibit a functional (motility and morphology) difference from each other that potentially skewed offspring sex ratio. Consistent with these findings, Kruger et al. (2019) also showed that complete deletion of the X-linked Slxl1 gene produced more male offspring by regulating post-meiotic germ cells transition (round spermatids to elongated spermatids).

An increased incidence of Y aneuploidy in spermatozoa was reported in another study, which selectively eliminated the Y spermatozoa and increased the proportion of X spermatozoa in mice and humans (Chaudhary et al., 2014). In accordance with this finding, we also reported that the viability of human Y spermatozoa is lower than that of X presumably due to the increased expression of apoptotic proteins in the live Y cells under stressful conditions, *in vitro*, thus, subsequently leading to shifts in the Y-to-X ratio (You et al., 2017). These findings indicate that functional properties of X and Y spermatozoa differ under certain *in vivo*/*in vitro* conditions due to the transcription of specific genes in particular cell types subsequently leading to the altered sex ratio at birth. Therefore, several factors, particularly genetic and environmental factors or both may differentially affect the ratio of X and Y spermatozoa by making one sperm type more sensitive to the external stress than that of the other. The ratio of X and Y spermatozoa in several animal species along with the methods used for differentiating between the two sperm types are summarized in Table 1. For some responses, there is a significant difference in means, but the difference is so small as to be of little or no biological significance because the distributions overlap almost completely. This overlap, for example, is so great as to make the mean difference useless for sexing sperm.

**TABLE 1:** Summary of the ratio of mammalian X and Y spermatozoa.

		**Cells**			**Hybridization**	
**References**	**Species (spermatozoa)**	**analyzed**	**Methods**	**Outcome measure**	**efficacy**	**X:Y**
Han et al., 1993b	Human (21–45 years)	813,066	Multicolor FISH	Fluorescence microscopy	NM	1.07:1
	Mouse (6–8 weeks)	10,390				1.24: 1
Eisenberg et al., 2012	Human	NM	Multicolor FISH	Epifluorescence microscopy (Zeiss Axiophot)	NM	1:1.06
	Oligospermic man					1:1.03
Smith et al., 2004	Human (cryopreserved)	∼1000	Multicolor FISH	Epifluorescence microscopy (Zeiss Axiophot)	99	∼1:1
Hossain et al., 2001	Human	3300	Multicolor FISH	Fluorescence microscopy	>98	∼1:1
Recio et al., 2001	Human (18–47 years)	9944–10,250	Multicolor FISH	Epifluorescence microscopy (Zeiss Axiophot)	99	1:1.03
Szyda et al., 2000	Bull	2122				1.15: 1
Irving et al., 1999	Man with only sons (>3)	NM	Multicolor FISH	Leitz Laborlux Ploemopak fluorescence microscopy	NM	1.102:1 (NS)
	Man with only daughters (>3)					1:1.17 (NS)
Hassanane et al., 1999	Bull	>10,000	Multicolor FISH	Fluorescence microscopy	NM	∼1:1
Chandler et al., 1998	Bull	∼100,000	PCR	UV/VIS spectrophotometer		1≠1
Halder and Tutscheck, 1998	Human	4506	Multicolor FISH	Epifluorescence microscopy (Zeiss Axiophot)		1.18:1
Samura et al., 1997	Human	>6000	Multicolor FISH and Percoll separation	Epifluorescence microscopy (Zeiss Axiophot)	99.8	∼1:1.02
			Multicolor FISH and swim-up/glass wool method			∼1:1
Martin et al., 1996	Human (21–52 years)	∼5000	Multicolor FISH	Epifluorescence microscopy (Zeiss Axiophot)		1.02:1
Griffin et al., 1996	Human	>300,000	Multicolor FISH	Epifluorescence microscopy (Zeiss Axiophot)	NM	∼1:1
Spriggs et al., 1996	Human (27–39 years)	50,000	Multicolor FISH	Epifluorescence microscopy (Zeiss Axiophot)	98	XY
Chevret et al., 1995	Human	94,575	Multicolor FISH and Percoll separation	Epifluorescence microscopy (Zeiss Axiophot)	NM	1.03:1
Martin et al., 1995a	Human (21–52 years)	>10,000	Multicolor FISH	Epifluorescence microscopy (Zeiss Axiophot)	NM	1.02:1
Martin et al., 1995b	Lymphoma patient (human, 32 years)	>10,000	Multicolor FISH	Epifluorescence microscopy (Zeiss Axiophot)		∼1:1
Williams et al., 1993	Human	2544–3860	Multicolor FISH	Epifluorescence microscopy (Zeiss Axiophot)	99	1:1.01
Cui and Matthews, 1993	Human	233	PCR	Direct microscopy	93.1	1.06:1
Lobel et al., 1993	Human		qPCR			∼1:1
Goldman et al., 1993	Human	60,000	Multicolor FISH			∼1:1
Han et al., 1993a	Human	∼1263	Multicolor FISH	Epifluorescence microscopy (Leitz microscope)		∼1:1
Benet et al., 1992	Human	505	Leishman staining	Analyzing zona-free hamster oocytes		1.02:1

**FISH, fluorescence in situ hybridization; NM, not mentioned; qPCR, real-time polymerase chain reaction; NS, non-significant.**

### Shape and Size

Despite the immense advancement in the field of developmental biology research, the basic idea of the spermatozoal structure remains unclear. As such, it is also unclear whether X and Y spermatozoa vary in their shape and size. By direct microscopic examination, two distinct types of spermatozoa: one type with a small, round head (presumably Y spermatozoa) and other type with a comparatively larger, elongated head (presumably X spermatozoa) were proposed by the early studies (Shettles, 1960, 1961). Both X and Y spermatozoa possess identical autosomes and an X or a Y chromosome. Thus, the difference in the size of X and Y spermatozoa may be due to the variations between X and Y chromosomes. Nevertheless, several researchers have suggested that the size of a sperm is not exclusively associated with its chromosomal content and may also be associated with its cytoplasmic content, which may vary in a specific sperm population during spermatogenesis (Shannon and Handel, 1993; Lankenau et al., 1994; Cui, 1997). In addition, Hossain et al. (2001) suggested that variations in the cytoplasmic content of X and Y spermatozoa introduced by meiosis and/or spermatogenesis were greater than those introduced by the sex chromosomes itself.

Although the preliminary hypothesis that X and Y spermatozoa were different based on their size and shape (Shettles, 1960, 1961) was supported by other researchers (Cui and Matthews, 1993; Cui, 1997), they were refuted with forceful arguments by the findings of several recent studies that used more specific methods for differentiating between X and Y spermatozoa (Hossain et al., 2001; Grant, 2006; Zavaczki et al., 2006). In an important study, Carvalho et al. (2013) used atomic force microscopy (AFM) and demonstrated that no differences existed in the shape and size of bovine X and Y spermatozoa even though 23 structural features between X and Y spermatozoa were assessed. AFM is highly specific as it provides detailed three-dimensional information of cells and is suitable for imaging the cell surface. Therefore, it is tempting to speculate that no or non-significant differences exist in the shape and size of X and Y spermatozoa. Previous studies mostly used non-specific comparative methods such as identification of Barr bodies and F bodies, which have low sensitivity in differentiating between X and Y spermatozoa, thus making the findings of these studies (i.e., X spermatozoa are larger than Y spermatozoa) less reliable. The findings of different studies on the shape and size of X and Y spermatozoa are summarized in Table 2.

**TABLE 2:** Findings of several studies on the size and shape of X and Y spermatozoa of human and domestic animals.

		**Cells**		**Enrichment**	**Outcome**	
**References**	**Spermatozoa**	**analyzed**	**Parameters**	**technique(s)**	**measured**	**Main findings**
Carvalho et al., 2013	Nellore bull	400	Sperm head shape and size	Flow cytometry	Atomic force microscopy	No difference
Zavaczki et al., 2006	Healthy, oligozoospermic, and normozoospermic man	>2000	Sperm head, perimeter, long and short axis, long or short axis, and tail length.	FISH	Phase-contrast microscopy	No difference
Hossain et al., 2001	Human	3300	Head length and width and tail length	FISH	Fluorescence microscopy	No difference
		520	Cell size and diameter			No difference
Van Munster et al., 1999	Bull	>1298	Sperm head volume	Flow cytometry	DIC microscopy	X > Y
Geraedts, 1997	Human	NM	Sperm surface	Feulgen staining of the Y chromosome	Fluorescence microscopy	X (7%) > Y
Cui, 1997	Human	895	Length, head perimeter, and length of the neck and tail	PCR identification of the Y chromosome	Light microscopy	X > Y
Cui and Matthews, 1993		217				
Chandler et al., 1998	Hereford bull	2214	Head areas		DIC microscopy	X > Y
Landrum and Shettles, 1960; Shettles, 1960	Human	NM	Head size and nuclear morphology	Dried unstained sperm observed directly	Phase-contrast microscopy	X > Y

**FISH, fluorescence in situ hybridization; DIC, differential interference contrast; NM, not mentioned.**

### Motility and Swimming Pattern

Owing to the high demand of sex preselection in animal reproduction, several studies have attempted to differentiate between X and Y spermatozoa over the past decades. Several researchers have used different methods to evaluate sex selection based on sperm motility; however, the efficacy of these methods is debatable. Additionally, it is unclear whether Y spermatozoa move faster than X spermatozoa. As an example, if Y spermatozoa move faster than X spermatozoa, a man should have a son, with an almost zero chance of having a daughter. Spermatozoa start swimming during the epididymal transition (Chang, 1951). Human spermatozoa travel at the rate of up to 3000 μm/min (Smith and Braun, 2012); however, some spermatozoa move slowly at the rate of 1000/min. Thus, a 55 μm long spermatozoon efficiently covers 1000–3000 μm. During this journey, the morphology and chemotaxis of spermatozoa and ionic factors, protein phosphorylation (especially tyrosine), ATP, cyclic adenosine monophosphate, protein kinase-A (PKA), enzymatic factors, seminal plasma factors, and calcium ions present in spermatozoa play a dynamic role in keeping the spermatozoa motile (Kwon et al., 2014b; Rahman et al., 2017b, 2018). Simultaneously, certain fascinating physiological processes such as capacitation and the acrosome reaction occur in spermatozoa (Visconti, 2009; Battistone et al., 2013; Rahman et al., 2017b). The difference in the ability of the X or Y spermatozoon to respond to these factors and processes will make it more active and motile than the other sperm type. Human X spermatozoa comprises 2.8% more genetic material (DNA) than Y spermatozoa; this difference is 3–4.2% between the X and Y spermatozoa of domestic livestock (Hendriksen et al., 1996). Several researchers have concluded that the variation in DNA content between X and Y spermatozoa may affect their motility and swimming pattern (Johnson et al., 1989; Johnson, 1994), however, the results of these studies are not conclusive.

Ericsson et al. (1973) used albumin gradient method and demonstrated that human Y spermatozoa (stained with quinacrine fluorochrome) reached the bottom of the gradient before X spermatozoa. These researches claimed that their method could identify >85% Y spermatozoa, of which 90–95% were motile. This finding was the first evidence of the difference in the swimming behavior of X and Y spermatozoa. The albumin gradient method has several advantages over other sperm sex preselection (methods for sperm selection along with their reliability status have been summarized in Table 3); however, detection of Y spermatozoa using quinacrine fluorochrome staining, as performed by Ericsson et al. (1973) was later proven to be non-specific (Flaherty and Matthews, 1996; Cui, 1997), thus leading to inappropriate results. In another study, Sarkar et al. (1984) reported that human X spermatozoa move slower (angular velocity decrease) than Y spermatozoa in the flow stream, however, the movement of both cells are similar in the stationary fluid.

**TABLE 3:** Acceptability of various methods for distinguishing between X and Y spermatozoa based on the difference in their motility, swimming pattern, and DNA content.

	**Sample**	**Enrichment**		**Target**	**Sperm**	
**References**	**(spermatozoa)**	**techniques**	**Base of separation**	**sperm**	**sorted (%)**	**Reliability**
Erickson, 1976	Human	Discontinuous albumin gradients	Y sperm has higher forward velocity than X sperm	Y	85	Unreliable
Evans et al., 1975					50	Unreliable
Ross et al., 1975					50	Unreliable
Quinlivan et al., 1982					52–74	Unreliable
Brandriff et al., 1986					50	Unreliable
Ueda and Yanagimachi, 1987					36.0–59.1	Unreliable
Iizuka et al., 1987	Human	Percoll gradients	Different motility of X and Y sperms	X	94	Unreliable
Wang et al., 1994					55.1	Unreliable
Van Kooij and Van Oost, 1992					50	Unreliable
Check et al., 1989	Human	Swim-up method	Difference in swimming pattern	X	81	Unreliable
Han et al., 1993b				X	50	Unreliable
Lobel et al., 1993				X	41.9–56.7	Unreliable
Yan et al., 2006				X and Y	50	Unreliable
Johnson et al., 1993	Human	Flow cytometry	Difference in DNA mass	X and Y	X = 80, X = 75	Reliable
Johnson, 2000	Livestock				X = 90	Reliable
Umehara et al., 2019	Mice	Swim-up method	TLR7/8 ligand activation		Y = 90 X = 81	Reliable

The controversy regarding the motility of X and Y spermatozoa was mainly provoked in 1998, when Penfold and coworker described their finding using flow cytometry and computer-assisted sperm analysis (CASA) for differentiating X and Y spermatozoa and measuring their motility parameters, respectively. They demonstrated that bull Y spermatozoa could not swim faster than X spermatozoa in a simple salt solution (Penfold et al., 1998). In accordance with the aforementioned findings, Alminana et al. (2014) reported a non-significant difference in the motility of X and Y spermatozoa. These findings indicate that no evidence is available that can help conclude whether Y spermatozoa are faster than X spermatozoa. This paradox becomes even more complex after considering the effect of oviductal fluid on the motility of X and Y spermatozoa (Zhu et al., 1994). In contrast, it has been confirmed that motility of X and Y spermatozoa vary under the certain condition *in vitro* (and presumably *in vivo*). For example, low pH, high temperature, and increased oxidative stress retarded motility in Y spermatozoa, whereas motility of X spermatozoa rapidly declined when spermatozoa are incubated in a high-pH condition (Shettles, 1970; Oyeyipo et al., 2017). In a recent study, Umehara et al. (2019) reported that ligand activation of TLR7/8 significantly decreased the motility of X spermatozoa (by altering ATP production) than that of Y. In addition, using the KO mice model, Rathje et al. (2019) reported that Yqdel males (XYRIIIqdel) produced less motile Y spermatozoa compared to X.

### Viability

Sperm viability is the ability of spermatozoa to sustain an intact plasma membrane and acrosomal membrane and to survive during passage through the oviduct in order to reach and fertilize the egg. Shettles (1960) suggested that X spermatozoa were stronger and more robust than Y spermatozoa because they had higher DNA content than Y spermatozoa. This preliminary hypothesis is supported by other investigators (Cui and Matthews, 1993; Flaherty and Matthews, 1996; Carvalho et al., 2013). Carvalho et al. (2013) reported that in addition to higher DNA content, larger size and longer length of the X chromosome made X spermatozoa more viable than Y spermatozoa. Recently, we demonstrated that when spermatozoa were incubated at different temperatures/culture conditions (You et al., 2017) or in a media containing 2,3,7,8-tetrachlorodibenzo-p-dioxin (an endocrine disruptor) (You et al., 2018), the Y spermatozoa represent a compromised viability compared to X. Moreover, the similar effects of other endocrine disruptors, such as dibromochloropropane and diazinon (Diaz) on the viability of Y spermatozoa were reported in another study (Song et al., 2018). The decreased viability of Y spermatozoa was mostly associated with the increased expression of apoptotic proteins in live Y spermatozoa (You et al., 2017), which subsequently affects the overall lifespan (You et al., 2018).

In particular, viability of spermatozoa is also related to female investment. The environment in the female reproductive tract (mostly fluid composition, pH and ionic concentration, and transcriptomic responses) affects the viability of X and Y spermatozoa and helps in selecting the best spermatozoa for fertilization (Dominko and First, 1997; Holt and Fazeli, 2010). Van Dyk et al. (2001) performed *in vitro* experiments mimicking the *in vivo* setting in the female reproductive tract and reported that Y spermatozoa survived for a longer duration than X spermatozoa, and that Y spermatozoa were more proficient to bind with zona pellucida than X spermatozoa (binding ratio, Y:X = 1.15:1.02). Other studies have suggested that higher expression of certain proteins (such as those involved in energy metabolism, e.g., ATP synthase subunit) provides more energy to Y spermatozoa, thus increasing their viability (Chayko and Martin-Deleon, 1992; Aranha and Martin-Deleon, 1995; Hendriksen, 1999; Chen et al., 2012). Based on the aforementioned findings, two different hypotheses can be drawn: (1) due to higher DNA content, X spermatozoa are more stable/viable than Y spermatozoa at least in the *in vitro* condition or (2) certain properties of Y cells may ensure that their prolonged viability in the female reproductive tract (*in vivo*) subsequently affects the lifespan of both cells in a distinct manner.

### Electrophobicity

Identification of subtle differences between X and Y spermatozoa is the only way to assess the preselection of a baby’s sex. Various studies have attempted to identify the difference in the electrical charge between X and Y spermatozoa. Epididymal epithelium secretes sialic acid (glycoprotein) that provides a net negative surface charge to the spermatozoa (Hoffmann and Killian, 1981). The difference in cell surface charge between the two sperm types is due to the difference in their exposed sialic acid content (Kaneko et al., 1984). These findings suggest that X and Y spermatozoa may exhibit differences in their electrophobicity. Results of free-flow electrophoresis indicated that the electrophoretic mobility of human X spermatozoa was higher than that of Y spermatozoa, suggesting that X spermatozoa exhibited higher negative charge than Y spermatozoa (Kaneko et al., 1984; Kaneko et al., 1993). In contrast, Engelmann et al. (1988) reported that human spermatozoa differentiated into X and Y fractions as they moved toward the anode, with the faster-moving and slower-moving fractions mainly comprising Y and X spermatozoa, respectively. The findings of Engelmann et al. (1988) were supported by those of another research group that used bovine spermatozoa (Blottner et al., 1994). The major limitation of these studies was the use of non-specific and unreliable quinacrine fluorescent staining to identify Y spermatozoa (F bodies) (Windsor et al., 1993), which led to inappropriate results. Recently, Ainsworth et al. (2011) observed that the use of CS-10 electrophoretic sperm isolation device did not skew the ratio of X and Y spermatozoa after their PCR-based differentiation. In particular, the device only isolated functional spermatozoa but was unable to specify their genotype. Thus, the movement of spermatozoa toward the anode might mainly depend on their surface sialic acid content, which allows them to comigrate with other spermatozoa during electrophoresis. Therefore, the findings of Ainsworth et al. (2011) clarified the unclear findings of the previous studies that reported a considerable difference in X and Y spermatozoa based on their electrophobicity.

### pH Susceptibility

Mammalian spermatozoa are immotile in the testis and become motile in response to several external factors that are initiated during their transfer through the epididymis. Of these factors, ionic concentration, particularly pH, plays an integral role in regulating the functional maturation of the spermatozoa. During sperm storage in the cauda epididymis, a slightly acidic pH is maintained. In the domestic animals, this acidic pH in the cauda epididymis inhibits sperm motility (Hamamah and Gatti, 1998). The association between pH and sperm functions becomes more complicated once the spermatozoa are released into the female reproductive tract. An equilibrium is required between the pH of the medium/female reproductive tract and intracellular pH of spermatozoa for successful fertilization (Blomqvist et al., 2006). In this section, we will discuss whether X and Y spermatozoa present differential pH susceptibility.

The preliminary findings indicated that X spermatozoa are larger and stronger than Y spermatozoa, suggesting that they are more stable in an acidic pH than Y spermatozoa (Landrum and Shettles, 1960; Shettles, 1960). Limited studies have supported this preliminary hypothesis. Muehleis and Long (1976) reported that insemination of an ovulated female rabbit with semen diluted with buffers of pH 5.4, 6.9, and 9.6 produced 48, 63, and 49% male offspring, respectively. This result partly supports Shettles’ hypothesis, which states that acidic pH (5.4) has deleterious effects on Y spermatozoa, thus affecting the probability (low probability of 48%) of conceiving male offspring; however, it is unclear whether an alkaline pH of 9.6 decreased the percentage (49%) of male offspring conceived particularly in comparison with the spermatozoa diluted with a buffer at pH 6.9. Pratt et al. (1987) reported a significant negative correlation between the vaginal pH and percentage of male offspring conceived in golden hamsters. Diasio and Glass (1971) reported that human X and Y spermatozoa could not be differentiated based on their pH affinity during their passage through a capillary tube containing media of varying pH. By examining 58489 human spermatozoa, recently we demonstrated that incubation of human spermatozoa in different pH conditions, including 6.5, 7.5, and 8.5 for 0–5 days were incapable in altering the ratio of Y:X chromosome (You et al., 2017). Thus, majority of the recent findings do not provide any logical explanation for X and Y spermatozoa acting differently at various pH conditions.

### Surface Properties (HY Antigen)

HY antigen is a male tissue-specific antigen. It is a fundamental part of the membrane of most male cells and is a specific antigen that controls the Y sperm-specific genes (Ohno and Wachtel, 1978). Here, we report evidence for the hypothesis that X and Y spermatozoa can be differentiated based on their surface HY antigen content.

Since the identification of a Y-linked histocompatibility antigen, scientists have believed that an immunological approach can be considered to control the sex ratio in mammals. This was initially demonstrated in a study by Bennett and Boyse (1973). They reported that the sex ratio of male offspring is significantly decreased (45.4%) when the female mice are inseminated with the spermatozoa treated with an anti-HY antibody compared with the untreated spermatozoa (53.4%). This study supported the hypothesis that the HY antigen could be used to distinguish between the X and Y spermatozoa; however, a minor shift in the male sex ratio after insemination with spermatozoa treated with the anti-HY antibody indicated only a small difference in the concentration of HY antigen between the two sperm types. Krco and Goldberg (1976) performed a 2-step cytotoxicity assay and identified the HY antigen in 8-celled mouse embryos, thus providing additional evidence of Y-chromosome expression of the HY antigen. Similar findings were obtained by other researchers by using several laboratory and domestic animal models (Silvers and Wachtel, 1977; Utsumi et al., 1993). Nevertheless, some researchers have found that the anti-HY antibody does not specifically bind to Y spermatozoa (Hoppe and Koo, 1984; Hendriksen et al., 1993; Sills et al., 1998). Sills et al. (1998) reported that the anti-HY antibody also binds to X spermatozoa and thus cannot be used to differentiate between X and Y spermatozoa. Besides, studies involving significant sex differentiation of human spermatozoa by using the surface antigen did not provide conclusive results (Jeulin et al., 1982; Sills et al., 1998).

## Molecular Insights of X and Y Spermatozoa

This section compares the molecular characteristics and biological activities of X and Y spermatozoa, with a special emphasis on the differences in their stress response, chromosomal abnormalities, and genomic/proteomic content.

### Response to Stress

Several studies have examined the etiology of male infertility in the context of oxidative stress (Agarwal et al., 2014) and physical, environmental, and occupational stress (Aitken, 2014; Barazani et al., 2014). Spermatozoa are the first cells that presented stress response (Gharagozloo and Aitken, 2011). In the MEDLINE database, the term “oxidative stress” has been mentioned in over 200,000 articles published between 2001 and to date, of which >1800 articles have focused on spermatozoa. Mechanisms underlying the response of X and Y spermatozoa during stress remain unclear. As X and Y spermatozoa differ in their genetic content, their response to stress may differs. Alminana et al. (2014) reported a non-significant difference while generating intracellular reactive oxygen species (ROS) by mitochondrial DNA in X and Y spermatozoa, and concluded that the tiny variations in DNA content between X and Y spermatozoa are unable to respond to stress differentially. A similar conclusion was drawn by other researchers (Ward and Coffey, 1991).

Mammalian spermatozoa cannot fertilize the oocyte before they are appropriately conditioned in the female reproductive tract even though they are motile and morphologically normal (Kwon et al., 2014b; Rahman et al., 2017b, 2019). Different parts of the female reproductive tract, such as the uterus, uterotubal junction, and oviduct, are specifically programmed to select only a functionally mature spermatozoon for fertilization (Holt and Fazeli, 2010). Once the spermatozoa reach the oviduct, they temporarily attach to the isthmus epithelium to undergo capacitation before ovulation (Rahman et al., 2015, 2016). Capacitation is a process during which complex molecular, biochemical, and physiological changes occur in spermatozoa in the female reproductive tract or in *in vitro* specialized media and is a prerequisite for fertilization (Salicioni et al., 2007; Visconti, 2012; Kwon et al., 2015). Therefore, preincubation of spermatozoa before fertilization is essential as capacitation duration might differ between X and Y spermatozoa depending on their genetic composition. Perez-Crespo et al. (2008) reported that mouse X and Y spermatozoa were differentially affected by elevated temperature. Moreover, they demonstrated that female mice mated with male mice that were exposed to scrotal heat stress on the day of mating produce more female pups. Altered sex ratio (i.e., increased number of female offspring) was also observed when the bovine spermatozoa incubated at 40°C for 4 h were used for insemination compared with those incubated at 38.5°C (Hendricks et al., 2009). Similarly, Lechniak et al. (2003) reported a significant increase in female blastocysts when bovine spermatozoa were preincubated for 24 h. In accordance with these findings, recently using an *in vitro* experimental design, we also demonstrated that human Y spermatozoa are more susceptible to stress then X *in vitro*, induced by variation of culture condition (You et al., 2017). In contrast, Iwata et al. (2008) reported that incubation of bovine spermatozoa with hyaluronic acid for 1 and 5 h produced 56.4 and 67.3% male embryos, respectively, thus skewing the expected 1:1 ratio. Therefore, it can be hypothesized that exposure of spermatozoa to external stress results in their differential survival; however, it is unclear whether stress provides selective survival advantage to X or Y sperms.

Recent studies have reported alterations in the sex ratio of human offspring exposed to increased levels of environmental chemicals, specifically endocrine-disrupting chemicals (EDs) (Van Larebeke et al., 2008; Mcdonald et al., 2014; Song et al., 2018; You et al., 2018). EDs interfere with the hormone biosynthesis and metabolism and may affect cellular physiology and reproduction (Anway et al., 2005). Mocarelli et al. (2000) reported that an increase in the concentration of 2,3,7, 8-tetrachlorodibenzo-*p*-dioxin (TCDD or dioxin) in the paternal serum elevated the probability of female births. Exposure of mice spermatozoa to TCDD *in vitro* also decreased the viability of Y spermatozoa (You et al., 2018), by potentially altering the embryonic male to female ratio. These findings were in accordance with another study (Ryan et al., 2002), where increased female births to men are documented following exposed to significantly high levels of TCDD. A similar effect of different EDs has been reported by several studies on humans and animals (Garry et al., 2002; Ikeda et al., 2005; Ishihara et al., 2007; Terrell et al., 2011). Despite few exceptions, for example, exposure to polychlorinated biphenyl was associated with an increase in the male births (Bonefeld-Jorgensen et al., 2001), the majority of the findings suggest that men exposed to a stressful environment are more likely to have girls (XX) than boys (XY) due to the higher DNA content in X spermatozoa than that in Y spermatozoa. Nevertheless, the particular stress response machineries between the two cell types remain unclear and need further investigation.

### Difference in Chromosomal Content of X and Y Spermatozoa

Molecular characteristics of spermatozoa including the chromosomal content/abnormalities play a pivotal role in inducing infertility (Pang et al., 1999; Schmidt et al., 2000). Briefly, chromosomal abnormality is defined as the loss of or presence of an extra or irregular portion of a chromosome that results in atypical number of chromosomes or a structural abnormality in one or more chromosomes (Jurewicz et al., 2014). In general, chromosomal abnormalities in embryos are thought to be acquired from eggs (Hassold et al., 1996), however, these abnormalities in spermatozoa may also substantially affect the embryos (Tesarik and Mendoza, 1996; Bonduelle et al., 2002). Abnormalities in the sex chromosomes contribute to >5% of major chromosomal errors in embryos, with ∼80% cases being of paternal origin (Hassold et al., 1996; Hassold and Hunt, 2001). In’t Veld et al. (1995) and Hoegerman et al. (1995) were the first to report an increased risk of *de novo* chromosomal errors, particularly in the sex chromosomes, in spermatozoa. The frequency of sex chromosome aneuploidy in healthy human spermatozoa is 0.13–1.20% (Egozcue et al., 1997). Templado et al. (2005) reviewed 23 studies and found that the average sex chromosome disomy (presence of an extra chromosome in a haploid state) in human spermatozoa was 0.26%. Of the 23 chromosomes in human spermatozoa, chromosomes 13, 18, 21, X, and Y are important because higher incidence of abnormalities in these chromosomes can to lead miscarriages or live births (Pang et al., 1999, 2005, 2010; Rubio et al., 2001). In this section, we review the evidence of sex chromosome abnormalities or aneuploidy, which leads to male reproductive dysfunction.

A combination of recently developed FISH and multicolor chromosome-specific probes can be used to investigate the chromosomal content of spermatozoa in order to establish a relative aneuploidy rate (Chevret et al., 1995). A higher percentage of sex chromosomal aneuploidy has been reported in oligoasthenoteratozoospermic patient spermatozoa compared to the autosomal aneuploidy in same individual, as well as sex chromosomal aneuploidy in healthy Y spermatozoa (Pang et al., 1999, 2010). In accordance with the aforementioned finding, Van Opstal et al. (1997) reported significantly higher errors in chromosomes X and Y than in chromosome 18 (autosome) in spermatozoa of azoospermic patients. In contrast, Pfeffer et al. (1999) reported higher incidence of chromosome 18 aneuploidy (0.7–10%) than sex chromosome aneuploidy (0–4.3%) in the swim-up sperm fraction of 10 infertile men with severe oligoasthenoteratozoospermia. Interestingly, the same study also reported higher sex chromosome aneuploidy, however, the aneuploidy was observed in the entire sperm pellet (Pfeffer et al., 1999). Therefore, different methods of sperm enrichment might also influence the incidence of aneuploidy.

Several studies have investigated the incidence of aneuploidy in X and Y chromosomes in human spermatozoa (Chevret et al., 1995; Martin et al., 1995a, b, 1996). Chevret et al. (1995) reported comparatively higher incidence of disomy in the X chromosome (0.04%) than that in the Y chromosome (0.009%) in normal male interphase spermatozoa, however, other studies have reported minute differences in the incidence of disomy in the X and Y chromosomes (Martin et al., 1995a, b; Samura et al., 1997). In contrast, Williams et al. (1993) reported higher incidence of disomy in the Y chromosome (YY, 0.11%) than that in the X chromosome (XX, 0.08%). This finding was further supported by another study that presented 0.18% (YY) and 0.07% (XX) disomy in the Y and X chromosomes, respectively (Martin et al., 1996). The difference between the reported aneuploidy rate in X and Y chromosomes remains unclear even though aneuploidy was detected using similar methods (i.e., 3-color FISH coupled with chromosome-specific probes and epifluorescence microscopy) in all the cases. Therefore, difference in the X and Y spermatozoa based on the frequency of aneuploidy in X and Y chromosomes remains unclear, which is in accordance with the other reported differences between these sperm types.

Recent studies have reported that exposure to certain EDs and pesticides induce sex chromosome abnormalities in spermatozoa (Smith et al., 2004; Xia et al., 2005; Perry, 2008). Epidemiological study revealed a significant association between exposure to two organochlorine chemicals and sex chromosome disomy in the spermatozoa collected from men who underwent infertility assessment at the Massachusetts General Hospital between January 2000 and May 2003 (Mcauliffe et al., 2012). They observed that higher serum levels of p,p’-dichlorodiphenyldichloroethylene (p,p’-DDE) significantly increased the frequency of XX (X sperm disomy), XY, and total sex chromosome disomy. Interestingly, men with higher serum levels of polychlorinated biphenyls (PCBs) presented a significant increase in the frequency of YY (Y sperm disomy), XY, and total sex chromosome disomy, however, this study did not provide further explanation of their findings, specifically on the mechanism by which the increased exposure to PCBs exerted protective effects against XX disomy and that in which the increased exposure to p,p’-DDE increased XX disomy. Therefore, possible mechanism(s) underlying the association between exposure to toxic chemicals, including EDCs (for example PCBs and p, p’-DDE), and sex chromosome disomy should be investigated. Moreover, similar epidemiological studies are warranted to identify the effects of various environmental chemicals and their association with chromosomal aberrations in the spermatozoa.

### Genomic and Proteomic Contents of X and Y Spermatozoa

Identification and quantification of genes/proteins in a cell provides fascinating insights regarding their cellular functions. Genomics deals with the structure, function, evolution, and mapping of genomes (Bader et al., 2003), whereas proteomics involves novel approaches for characterizing proteins by performing qualitative and quantitative analyses (Rahman et al., 2016, 2017a, 2018). A spermatozoon provides half of the nuclear genetic material to the diploid offspring via fertilization. Thus, examination of the genes and protein content in spermatozoa might provide potential insights on their functions. It has been reported that haploid spermatids are capable of active chromosomal (including sex chromosomes) transcription important for their growth and survival (Braun et al., 1989). As X and Y chromosome-bearing-spermatids express distinct genes encoded by each sex chromosome (Hendriksen, 1999), it might result in the proteomics difference between X and Y spermatozoa. Although the majority of the genes are shared between X and Y spermatids via the intracellular bridge (Braun et al., 1989), complete sharing has not occurred for all gene products (Hendriksen, 1999). Therefore, X and Y spermatozoa can be differentiated based on their gene/protein content. In this section, we review studies on the genomic and proteomic characteristics of X and Y spermatozoa and have elucidated their association with the morphophysiological characteristics of the two sperm types.

To date, very limited studies have identified and characterized genes that are differentially expressed in X and Y spermatozoa. Spermatozoa contain a minute amount of total RNA (human spermatozoon, 0.015 pg; bovine spermatozoon, 1.8 × 10^–4^ pg) compared to that in the somatic cells (1–3 pg). This small amount of RNA per spermatozoon is the major drawback for research on gene expression in these cells. Chen et al. (2014) used comprehensive genomic approaches and identified 31 differentially expressed genes in bovine X and Y spermatozoa (27 and 4 genes upregulated in X and Y spermatozoa, respectively). Using the RNA sequencing technology, it has been reported that the X chromosome encodes 492 genes, whereas the Y chromosome encodes only 15 genes in mouse spermatozoa. Some of these genes (particularly receptors) are also shown to be related to the growth, survivability, and functions of specific sperm types (Umehara et al., 2019). Therefore, differentially expressed genes might help in identifying the genetic background of stable differences between X and Y spermatozoa. Alminana et al. (2014) observed that spermatozoa revealed sex-specific gene expression in the oviduct of female pigs inseminated with either X or Y spermatozoa. When insemination was performed using Y spermatozoa, 271 transcripts were downregulated and 230 transcripts were upregulated in the oviduct. Thus, the oviduct might have special biological sensors for screening spermatozoa. Bermejo-Alvarez et al. (2010) reported significant differences in the mRNA levels of *GSTM3*, *DNMT3A*, and *PGRMC1* between bovine blastocysts produced by *in vitro* fertilization with X spermatozoa and those produced by *in vitro* fertilization with Y spermatozoa. This indicates that the oocyte might also regulate an identical mechanism for reorganizing the different spermatozoa. Recent advances in genomic studies have provided several improved techniques that allow complete lysis of spermatozoa and isolation of total RNA (Kirley, 1990; Meng and Feldman, 2010; Chen et al., 2014). Therefore, further studies are warranted to identify the genes expressed in the sexed spermatozoa of different species.

Mature spermatozoa undergo minimal transcription (there are few ribosomes, so translation is not possible) as well as protein synthesis (Kwon et al., 2014a, 2015). Therefore, these cells are extremely suitable for performing proteomic analysis. Direct comparison of protein levels in various cells can identify the markers responsible for differences between these cells (Park et al., 2013; Kwon et al., 2014a). Literature searches indicated that limited studies have been performed to evaluate the proteomic blueprint of X and Y spermatozoa to date. Hendriksen et al. (1996) reported a non-significant difference in the concentration of plasma membrane proteins in porcine X and Y spermatozoa. This study indicated that sexing of spermatozoa cannot be performed based on their surface properties. Chen et al. (2012) used two-dimensional electrophoresis along with mass spectrometry (2DE-MS/MS) and identified 42 differentially expressed proteins between X and Y spermatozoa. Of these, 11 proteins were upregulated and 4 were downregulated in X spermatozoa compared with those in the Y spermatozoa (*P* < 0.05). This finding was partly supported by other investigators (De Canio et al., 2014; Scott et al., 2018). Using label-free shotgun nUPLC-MS/MS De Canio et al. (2014) found that 15 and 2 proteins were upregulated in X and Y spermatozoa, respectively. In another recent study, Scott et al. (2018) identified the differential expression of eight proteins between X- and Y-bearing spermatozoa. Of these, the protein related to the embryo development (EF-hand domain-containing protein 1) was expressed abundantly in the Y spermatozoa, whereas majority of other detected proteins were abundant in the X spermatozoa. Since abundant proteins in Y spermatozoa help in post-fertilization embryo development and further in the survivability of male baby over female, which also support lightly higher males (105) than female (100) babies at birth. Despite differential expression of particular proteins between the two cell types, zinc ion binding structure of bovine heart cytochrome c (2EIN_R) is the only protein reported by Chen et al. (2012) with the characteristics unique expression in only X spermatozoa. Therefore, 2EIN_R could be considered as a novel biomarker for differentiating the two cell types/sex preselection purpose. In contrast, majority of these proteomics studies identified limited identical proteins despite the samples being collected from the same animal species (bull). Moreover, Chen et al. (2012) reported increased levels of tubulin isoforms α3 and β4B in X spermatozoa. In contrast, De Canio et al. (2014) reported different expression profiles of two tubulin isoforms α8 and β2B. Use of different proteomic approaches (i.e., 2DE-MS/MS, nUPLC-MS/MS, and SWATH-MS analysis) in these studies might have led to these differences. Based on these findings, it is essential to speculate that X and Y spermatozoa can at least be different based on their protein content; however, further studies are warranted to identify the validated markers that could differentiate these two cell types appropriately. In addition, proteomic analysis of X and Y spermatozoa from different animal species should be conducted for their practical application particularly for immunosexing techniques.

**FIGURE 2:**
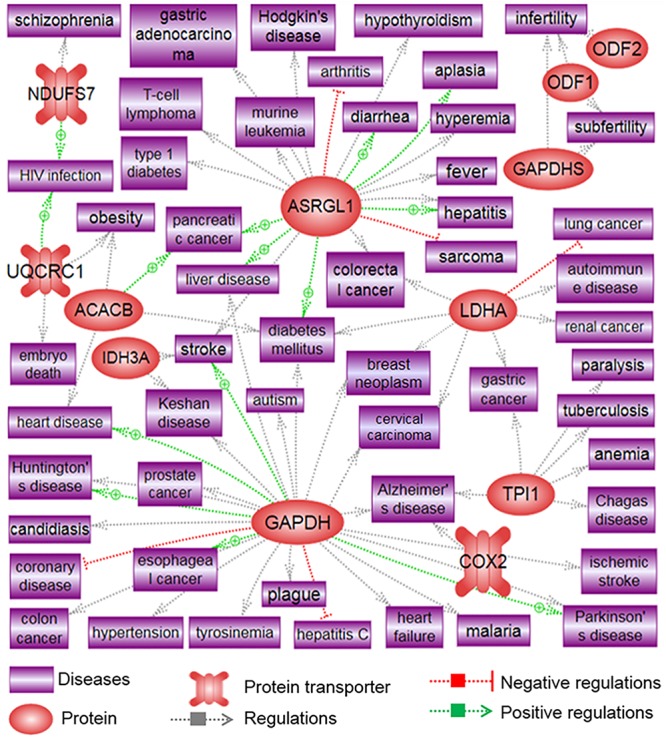
Signaling pathways associated with highly differentiated proteins in X spermatozoa. The illustration was prepared using Pathway Studio (Elsevier^®^, Ariadne Genomics, Inc.) after performing a literature search in the PubMed database.

**FIGURE 3:**
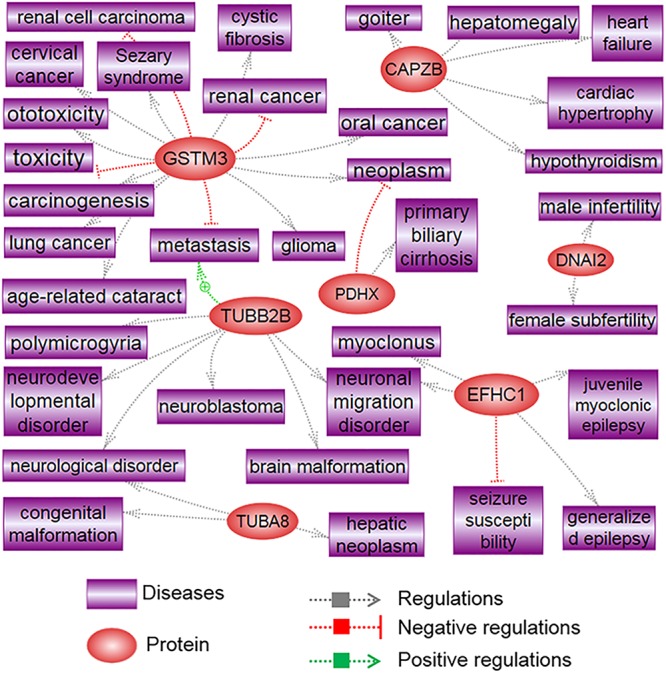
Signaling pathways associated with highly differentiated proteins in Y spermatozoa. The illustration was prepared using Pathway Studio (Elsevier^®^, Ariadne Genomics, Inc.) after performing a literature search in the PubMed database.

Proteins that are differentially expressed in X and Y spermatozoa are summarized in Table 4 (data collected from published studies). We used the Pathway Studio program and found that proteins that were highly expressed in X spermatozoa were significantly (*P* < 0.05) correlated with five major canonical pathways/signaling, whereas proteins that were highly expressed in Y spermatozoa were correlated with four pathways/signaling (Table 4). The differences in the protein content and associated signaling pathways between X and Y spermatozoa might provide a theoretical basis to distinguish between these sperm types. Nevertheless, it is uncertain whether these differences are correlated with the biological aspect of X and Y spermatozoa. By using the same program, we determined the associated disease processes that were regulated by the differentially expressed proteins in X and Y spermatozoa. By using this simple illustration (Figures 2, 3), one may have a hypothetical presumption regarding the occurrence of specific diseases in men and women. For example, L-lactate dehydrogenase A and testis-specific glyceraldehyde 3-phosphate dehydrogenase, which are highly expressed in X spermatozoa, are found to be functionally associated with breast neoplasm and cervical carcinoma (Figure 2). Both the cancers are the leading cause of cancer deaths in women (Siegel et al., 2015). In accordance, epidemiological investigation in humans revealed relatively higher incidence of anemia (Malhotra et al., 2004; Alvarez-Uria et al., 2014), Alzheimer’s disease (Vina and Lloret, 2010), Huntington’s disease (Panas et al., 2011), and trypanosoma (Pepin et al., 2002) in women. These diseases were also found to be associated with proteins that were highly expressed in X spermatozoa (Figure 2). Similarly, abundant proteins in Y spermatozoa, that is TUBA8 and GSTM3, were found to be associated with hepatic cancer and renal cancer, respectively, and the prevalence of both diseases were reported to be high in men compared to the women (Woldrich et al., 2008; Wu et al., 2018). However, few other diseases that are found to be related with the differentially expressed proteins either in the X and Y spermatozoa represent different results compared to the epidemiological data (Figure 3). For example, heart failure was found to be related with CAPZB that was highly expressed in Y spermatozoa, however, its incidence is lower in men than that in women. Consistently, tuberculosis was found to be related with the altered functionality of TPI1 that was more highly expresses in X spermatozoa than in Y spermatozoa. However, the incidence of this disease is high in men than women. These inconsistencies presumably due to the Pathway Studio program, generated protein pathways by using information present in the PubMed database, which are incapable to explain every disease condition precisely. In addition, despite the differential expression of a particular protein between two cell types, the existence of majority of the proteins is constant between them. Therefore, the increased expression of a protein in the particular cell may not always represent their functional activation. Another major drawback of this hypothesis is that minor proteomic alterations (<2-fold) between X and Y spermatozoa may not necessarily display any significant differences in protein expression in the resulting offspring, and thus could predispose alternative sconclusion.

**TABLE 4:** List of differentially expressed proteins in X and Y chromosome bearing spermatozoa.

	**Accession**	**X/Y**	**Proteomic**	**Related pathways**	
**Proteins (symbol)**	**no**	**(intensity)**	**technique**	**(*P* > 0.05)**	**References**
**Upregulated proteins in X spermatozoa**					
Seminal plasma protein PDC-109 (BSP1)	P02784	1.92	nUPLC-MS/MS		De Canio et al., 2014
Glyceraldehyde 3-phosphate dehydrogenase (GAPDH)	P10096	1.69		Glucose metabolism, glycolysis	
Outer dense fiber protein 2 (ODF2)	Q2T9U2	1.63			
Tubulin beta 4A (TUBB4A)	Q3ZBU7	1.58			
L-Lactate dehydrogenase A (LDHA)	P19858	1.56		Glucose metabolism	
Outer dense fiber protein 1 (ODF1)	Q29438	1.53			
A kinase anchor protein 3 (AKAP3)	O77797	1.51			
L-Asparaginase (ASRGL1)	Q32LE5	1.44			
Tubulin beta 4B (TUBB4B)	Q3MHM5	1.42			
Tubulin alpha 3 (TUBA3E)	Q32KN8	1.42			
Outer dense fiber protein 3 (ODF3)	Q2TBH0	1.42			
Glyceraldehyde 3-phosphate dehydrogenase, testis specific (GAPDHS)	Q2KJE5	1.40		Glucose metabolism	
Sperm acrosome membrane associated protein 1 (SPACA1)	Q2YDG7	1.36			
Triosephosphate isomerase (TPI1)	Q5E956	1.36		Glucose metabolism, mTOR signaling	
Calmodulin (CALM)	P62157	1.36			
FUN14 domain-containing protein 2 (FUNDC2)	NP_776763	2.612	SWATH-MS		Scott et al., 2018
Acetyl-CoA carboxylase, type beta (ACACB)	CAI84638	2.149			
NADH dehydrogenase [ubiquinone] iron-sulfur protein 7, mitochondrial (NDUFS7)	NP_001033111	1.502			
Sorting and assembly machinery component 50 homolog (SAMM50)	NP_001040088	1.491			
Cytochrome c oxidase subunit 2 (COX2)	QBH99117.1	1.399		mTOR signaling, TCA cycle, oxidative phosphorylation	
Protein FAN	NP_001179158	2.72	MALDI-TOF-MS		Chen et al., 2012
Oxidase heme a, cytochrome	771727A	1.71			
Cytochrome b–c1 complex subunit 1, mitochondrial (UQCRC1)	P31800	2.17		mTOR signaling, TCA cycle, oxidative phosphorylation	
3-Hydroxyisobutyrate dehydrogenase (HIBADH)	AAI05544	1.58			
Tubulin alpha-3 chain (TUBA3)	Q32KN8	1.68			
Isocitrate dehydrogenase [NAD] subunit alpha, mitochondrial (IDH3A)	P41563	1.83			
Chain A, the structure of crystalline profilin-beta-actin	2BTF_A	1.69			
A Chain A, episelection: Novel Ki ∼ nanomolar inhibitors of serine proteases	1BTW_A	1.8	LC-MS		
R Chain R, zinc ion binding structure of bovine heart cytochrome c	2EIN_R	Only in X			
Tubulin beta-4B chain (TUBB4B)	NP 001029835	1.51			
Isocitrate dehydrogenase 3 (NAD +) alpha (IDH3A)	AAI18260	1.50			
**Upregulated proteins in Y spermatozoa**					
Tubulin alpha 8 (TUBA8)	Q2HJB8	0.43	nUPLC-MS/MS	Guanylate cyclase, and notch	De Canio et al., 2014
Tubulin beta 2B (TUBB2B)	Q6B856	0.26			
EF-hand domain-containing protein 1 (EFHC1)	NP_001179173.1	0.05	SWATH-MS		Scott et al., 2018
Pyruvate dehydrogenase protein X component (PDHX)	NP_001069219.1	0.393			
Dynein intermediate chain 2, axonemal (DNAI2)	XP_027374681.1	0.457			
Chain A, crystal structure of bovine heart mitochondrial Bc1 with Jg144 inhibitor	2FYU_A	0.52	MALDI-TOF-MS		Chen et al., 2012
ATP synthase subunit beta, mitochondrial (ATP5B)	P00829	0.48			
F-actin-capping protein subunit beta (CAPZB)	P79136	0.50		Guanylate cyclase, notch, and actin cytoskeleton assembly	
lutathione *S*-transferase, mu 3 (brain) (GSTM3)	AAI12492	0.51	LC-MS	Glutathione metabolism	

**Differentially expressed proteins in X and Y sperms were entered in the Pathway Studio program (Elsevier®) to identify the significantly correlated (P < 0.05) signaling pathways in these sperms. Briefly, protein names (symbols) were entered into the program to determine significantly matching pathways for each differentially expressed protein based on the information extracted from the NCBI PubMed database. Signaling pathways associated with the differentially expressed proteins were confirmed using a PubMed Medline hyperlink that was embedded in each node. Fisher’s exact test was used to determine whether a pathway was statistically correlated with the target protein. P < 0.05 was considered as statistically significant.**

## Conclusion

Nature has developed many mechanisms to make genetically different sperm phenotypically identical within a male to avoid a fertilization advantage of one allele over another. Mendel’s law of independent assortment would not be true if some alleles had a fertilization advantage. An example (very rare) where different alleles affect fertility is the T allele system on Chromosome 17 in mice, in which great infertility occurs (Colaco and Modi, 2018). Among the mechanisms employed by nature are intercellular bridges of clutches of 32 or more spermatagonia and spermatids so that RNA and proteins are exchanged in the clutches of the developing sperm with different genotypes, thus homogenizing the cytoplasm, cell membranes, and so on. Another mechanism is extremely limited post-meiotic gene expression during spermatogenesis/spermiogenesis. The Sertoli nurse cells take over many essential cellular molecular functions during this period to compensate. Additional mechanism is coating sperm with surface molecules during epididymal maturation to make sperm look alike. These mechanisms explain why sperm are so identical, including X and Y sperm (within a male). Indeed, differentiation between X and Y spermatozoa has been of immense interest to researchers, physicians, and breeders, since the beginning of recorded history. Various methods have been used to distinguish between X and Y spermatozoa; however, the practical validity of these methods is questionable. The only consistent *de novo* difference identified between X and Y spermatozoon to date is in their DNA content, which might be responsible for the differential expression of some genes and proteins and the occurrence of certain diseases in a sex specific manner; however, it is unclear whether this difference in the DNA content results in other physical, chemical, and functional differences between X and Y spermatozoa. Moreover, the ambiguity in the existing findings might be due to the use of non-specific or less-specific methods for distinguishing between X and Y spermatozoa. Therefore, further studies using more specific, non-invasive (less injurious to cells) methods to distinguish between the two sperm types for the sex preselection of offspring are warranted.

## Author Contributions

MR and M-GP conceived the idea. MR drafted the manuscript and prepared the artworks. M-GP supervised the whole work. Both authors crucially revised the manuscript for important intellectual content and approved the final version to be published.

## Conflict of Interest

The authors declare that the research was conducted in the absence of any commercial or financial relationships that could be construed as a potential conflict of interest.

## References

[B1] Agarwal A. Sharma R. K. Sharma R. Assidi M. Abuzenadah A. M. Alshahrani S. (2014). Characterizing semen parameters and their association with reactive oxygen species in infertile men. *Reprod. Biol. Endocrinol.* 12:33. 10.1186/1477-7827-12-33 24885775 PMC4047553

[B2] Ainsworth C. J. Nixon B. Aitken R. J. (2011). The electrophoretic separation of spermatozoa: an analysis of genotype, surface carbohydrate composition and potential for capacitation. *Int. J. Androl.* 34 e422–e434. 10.1111/j.1365-2605.2011.01164.x 21564134

[B3] Aitken R. J. (2014). Age, the environment and our reproductive future: bonking baby boomers and the future of sex. *Reproduction* 147 S1–S11. 10.1530/REP-13-0399 24194569

[B4] Alminana C. Caballero I. Heath P. R. Maleki-Dizaji S. Parrilla I. Cuello C. (2014). The battle of the sexes starts in the oviduct: modulation of oviductal transcriptome by X and Y-bearing spermatozoa. *BMC Genomics* 15:293. 10.1186/1471-2164-15-293 24886317 PMC4035082

[B5] Alvarez-Uria G. Naik P. K. Midde M. Yalla P. S. Pakam R. (2014). Prevalence and severity of anaemia stratified by age and gender in rural India. *Anemia* 2014:176182. 10.1155/2014/176182 25614831 PMC4277798

[B6] Anway M. D. Cupp A. S. Uzumcu M. Skinner M. K. (2005). Epigenetic transgenerational actions of endocrine disruptors and male fertility. *Science* 308 1466–1469. 10.1126/science.1108190 15933200 PMC11423801

[B7] Aranha I. P. Martin-Deleon P. A. (1995). Mouse chromosome 6 in Rb translocations: consequences in singly and doubly heterozygous males. *Cytogenet. Cell Genet.* 69 253–259. 10.1159/000133975 7698024

[B8] Bader G. D. Heilbut A. Andrews B. Tyers M. Hughes T. Boone C. (2003). Functional genomics and proteomics: charting a multidimensional map of the yeast cell. *Trends Cell Biol.* 13 344–356. 10.1016/s0962-8924(03)00127-2 12837605

[B9] Barazani Y. Katz B. F. Nagler H. M. Stember D. S. (2014). Lifestyle, environment, and male reproductive health. *Urol. Clin. North Am.* 41 55–66. 10.1016/j.ucl.2013.08.017 24286767

[B10] Battistone M. A. Da Ros V. G. Salicioni A. M. Navarrete F. A. Krapf D. Visconti P. E. (2013). Functional human sperm capacitation requires both bicarbonate-dependent PKA activation and down-regulation of Ser/Thr phosphatases by Src family kinases. *Mol. Hum. Reprod.* 19 570–580. 10.1093/molehr/gat033 23630234 PMC3749807

[B11] Bean B. (1990). Progenitive sex ratio among functioning sperm cells. *Am. J. Hum. Genet.* 47 351–353. 2378363 PMC1683735

[B12] Benet J. Genesca A. Navarro J. Egozcue J. Templado C. (1992). Cytogenetic studies in motile sperm from normal men. *Hum. Genet.* 89 176–180. 1587528 10.1007/BF00217119

[B13] Bennett D. Boyse E. A. (1973). Sex ratio in progeny of mice inseminated with sperm treated with H-Y antiserum. *Nature* 246 308–309. 10.1038/246308a0 4586316

[B14] Bermejo-Alvarez P. Rizos D. Rath D. Lonergan P. Gutierrez-Adan A. (2010). Sex determines the expression level of one third of the actively expressed genes in bovine blastocysts. *Proc. Natl. Acad. Sci. U.S.A.* 107 3394–3399. 10.1073/pnas.0913843107 20133684 PMC2840439

[B15] Bibbins P. E. Jr. Lipshultz L. I. Ward J. B. Jr. Legator M. S. (1988). Fluorescent body distribution in spermatozoa in the male with exclusively female offspring. *Fertil. Steril.* 49 670–675. 10.1016/s0015-0282(16)59838-0 3350162

[B16] Blomqvist S. R. Vidarsson H. Soder O. Enerback S. (2006). Epididymal expression of the forkhead transcription factor Foxi1 is required for male fertility. *EMBO J.* 25 4131–4141. 10.1038/sj.emboj.7601272 16932748 PMC1560351

[B17] Blottner S. Bostedt H. Mewes K. Pitra C. (1994). Enrichment of bovine X and Y spermatozoa by free-flow electrophoresis. *Zentralbl. Veterinarmed. A* 41 466–474. 10.1111/j.1439-0442.1994.tb00113.x 7863737

[B18] Bonduelle M. Van Assche E. Joris H. Keymolen K. Devroey P. Van Steirteghem A. (2002). Prenatal testing in ICSI pregnancies: incidence of chromosomal anomalies in 1586 karyotypes and relation to sperm parameters. *Hum. Reprod.* 17 2600–2614. 10.1093/humrep/17.10.2600 12351536

[B19] Bonefeld-Jorgensen E. C. Andersen H. R. Rasmussen T. H. Vinggaard A. M. (2001). Effect of highly bioaccumulated polychlorinated biphenyl congeners on estrogen and androgen receptor activity. *Toxicology* 158 141–153. 10.1016/s0300-483x(00)00368-1 11275356

[B20] Brandriff B. F. Gordon L. A. Haendel S. Singer S. Moore D. H. II Gledhill B. L. (1986). Sex chromosome ratios determined by karyotypic analysis in albumin-isolated human sperm. *Fertil. Steril.* 46 678–685. 10.1016/s0015-0282(16)49648-2 3463512

[B21] Braun R. E. Behringer R. R. Peschon J. J. Brinster R. L. Palmiter R. D. (1989). Genetically haploid spermatids are phenotypically diploid. *Nature* 337 373–376. 10.1038/337373a0 2911388

[B22] Carvalho J. O. Silva L. P. Sartori R. Dode M. A. (2013). Nanoscale differences in the shape and size of X and Y chromosome-bearing bovine sperm heads assessed by atomic force microscopy. *PLoS One* 8:e59387. 10.1371/journal.pone.0059387 23527178 PMC3602057

[B23] Chandler J. E. Steinholt-Chenevert H. C. Adkinson R. W. Moser E. B. (1998). Sex ratio variation between ejaculates within sire evaluated by polymerase chain reaction, calving, and farrowing records. *J. Dairy Sci.* 81 1855–1867. 10.3168/jds.s0022-0302(98)75756-x 9710752

[B24] Chang M. C. (1951). Fertilizing capacity of spermatozoa deposited into the fallopian tubes. *Nature* 168 697–698. 10.1038/168697b0 14882325

[B25] Chaudhary I. Jain M. Halder A. (2014). Sperm sex ratio (X: Y ratio) and its variations. *Austin J. Reprod. Med. Infertil.* 1:7.

[B26] Chayko C. A. Martin-Deleon P. A. (1992). The murine Rb(6.16) translocation: alterations in the proportion of alternate sperm segregants effecting fertilization in vitro and in vivo. *Hum. Genet.* 90 79–85. 1427792 10.1007/BF00210748

[B27] Check J. H. Shanis B. S. Cooper S. O. Bollendorf A. (1989). Male sex preselection: swim-up technique and insemination of women after ovulation induction. *Arch. Androl.* 23 165–166. 10.3109/01485018908986839 2511816

[B28] Chen X. Yue Y. He Y. Zhu H. Hao H. Zhao X. (2014). Identification and characterization of genes differentially expressed in X and Y sperm using suppression subtractive hybridization and cDNA microarray. *Mol. Reprod. Dev.* 81 908–917. 10.1002/mrd.22386 25223630

[B29] Chen X. Zhu H. Wu C. Han W. Hao H. Zhao X. (2012). Identification of differentially expressed proteins between bull X and Y spermatozoa. *J. Proteomics* 77 59–67. 10.1016/j.jprot.2012.07.004 22820535

[B30] Chevret E. Rousseaux S. Monteil M. Pelletier R. Cozzi J. Sele B. (1995). Meiotic segregation of the X and Y chromosomes and chromosome 1 analyzed by three-color FISH in human interphase spermatozoa. *Cytogenet. Cell Genet.* 71 126–130. 10.1159/000134090 7656580

[B31] Clapham D. E. (2013). Sperm BerserKers. *Elife.* 2:e01469. 10.7554/eLife.01469 24137547 PMC3791457

[B32] Colaco S. Modi D. (2018). Genetics of the human Y chromosome and its association with male infertility. *Reprod. Biol. Endocrinol.* 16:14. 10.1186/s12958-018-0330-5 29454353 PMC5816366

[B33] Cui K. H. (1997). Size differences between human X and Y spermatozoa and prefertilization diagnosis. *Mol. Hum. Reprod.* 3 61–67. 10.1093/molehr/3.1.61 9239709

[B34] Cui K. H. Matthews C. D. (1993). X larger than Y. *Nature* 366 117–118. 8232551 10.1038/366117b0

[B35] Curtsinger J. W. Ito R. Hiraizumi Y. (1983). A two-generation study of human sex-ratio variation. *Am. J. Hum. Genet.* 35 951–961. 6614009 PMC1685814

[B36] De Canio M. Soggiu A. Piras C. Bonizzi L. Galli A. Urbani A. (2014). Differential protein profile in sexed bovine semen: shotgun proteomics investigation. *Mol. Biosyst.* 10 1264–1271. 10.1039/c3mb70306a 24226273

[B37] Diasio R. B. Glass R. H. (1971). Effects of pH on the migration of X and Y sperm. *Fertil. Steril.* 22 303–305. 10.1016/s0015-0282(16)38224-3 4102480

[B38] Dominko T. First N. L. (1997). Relationship between the maturational state of oocytes at the time of insemination and sex ratio of subsequent early bovine embryos. *Theriogenology* 47 1041–1050. 10.1016/s0093-691x(97)00061-7 16728054

[B39] Egozcue J. Blanco J. Vidal F. (1997). Chromosome studies in human sperm nuclei using fluorescence in-situ hybridization (FISH). *Hum. Reprod. Update* 3 441–452. 10.1093/humupd/3.5.441 9528909

[B40] Eisenberg M. L. Murthy L. Hwang K. Lamb D. J. Lipshultz L. I. (2012). Sperm counts and sperm sex ratio in male infertility patients. *Asian J. Androl.* 14 683–686. 10.1038/aja.2012.58 22842703 PMC3735000

[B41] Engelmann U. Krassnigg F. Schatz H. Schill W. B. (1988). Separation of human X and Y spermatozoa by free-flow electrophoresis. *Gamete Res.* 19 151–160. 10.1002/mrd.1120190205 3209178

[B42] Erickson J. D. (1976). The secondary sex ratio in the United States 1969-71: association with race, parental ages, birth order, paternal education and legitimacy. *Ann. Hum. Genet.* 40 205–212. 10.1111/j.1469-1809.1976.tb00182.x 1015815

[B43] Ericsson R. J. Langevin C. N. Nishino M. (1973). Isolation of fractions rich in human Y sperm. *Nature* 246 421–424. 10.1038/246421a0 4587152

[B44] Evans J. M. Douglas T. A. Renton J. P. (1975). An attempt to separate fractions rich in human Y sperm. *Nature* 253 352–354. 10.1038/253352a0 1110779

[B45] Flaherty S. P. Matthews C. D. (1996). Application of modern molecular techniques to evaluate sperm sex selection methods. *Mol. Hum. Reprod.* 2 937–942. 10.1093/molehr/2.12.937 9237237

[B46] Garry V. F. Harkins M. E. Erickson L. L. Long-Simpson L. K. Holland S. E. Burroughs B. L. (2002). Birth defects, season of conception, and sex of children born to pesticide applicators living in the Red River Valley of Minnesota, USA. *Environ. Health Perspect.* 110(Suppl. 3), 441–449. 10.1289/ehp.02110s3441 12060842 PMC1241196

[B47] Gellatly C. (2009). Trends in population sex ratios may be explained by changes in the frequencies of polymorphic alleles of a sex ratio gene. *Evol. Biol.* 36 190–200. 10.1007/s11692-008-9046-3

[B48] Geraedts J. P. (1997). X spermatozoa larger than Y in 1973. *Mol. Hum. Reprod.* 3 545–546. 10.1093/molehr/3.6.545 9239744

[B49] Gharagozloo P. Aitken R. J. (2011). The role of sperm oxidative stress in male infertility and the significance of oral antioxidant therapy. *Hum. Reprod.* 26 1628–1640. 10.1093/humrep/der132 21546386

[B50] Goldman A. S. Fomina Z. Knights P. A. Hill C. J. Walker A. P. Hulten M. A. (1993). Analysis of the primary sex ratio, sex chromosome aneuploidy and diploidy in human sperm using dual-colour fluorescence in situ hybridisation. *Eur. J. Hum. Genet.* 1 325–334. 10.1159/000472431 8081946

[B51] Grant V. J. (2006). Entrenched misinformation about X and Y sperm. *BMJ* 332:916. 10.1136/bmj.332.7546.916-b PMC1440662 16613983

[B52] Griffin D. K. Abruzzo M. A. Millie E. A. Feingold E. Hassold T. J. (1996). Sex ratio in normal and disomic sperm: evidence that the extra chromosome 21 preferentially segregates with the Y chromosome. *Am. J. Hum. Genet.* 59 1108–1113. 8900240 PMC1914829

[B53] Halder A. Tutscheck B. (1998). Analysis of meiotic segregation in human nondecondensed interphase spermatozoa by triple colour rapid direct fluorescent in situ hybridization. *Indian J. Med. Res.* 107 94–97. 9540284

[B54] Hamamah S. Gatti J. L. (1998). Role of the ionic environment and internal pH on sperm activity. *Hum. Reprod.* 13(Suppl. 4), 20–30. 10.1093/humrep/13.suppl_4.20 10091055

[B55] Han T. L. Flaherty S. P. Ford J. H. Matthews C. D. (1993a). Detection of X- and Y-bearing human spermatozoa after motile sperm isolation by swim-up. *Fertil. Steril.* 60 1046–1051. 10.1016/s0015-0282(16)56408-5 8243684

[B56] Han T. L. Ford J. H. Webb G. C. Flaherty S. P. Correll A. Matthews C. D. (1993b). Simultaneous detection of X- and Y-bearing human sperm by double fluorescence in situ hybridization. *Mol. Reprod. Dev.* 34 308–313. 10.1002/mrd.1080340311 8471253

[B57] Hassanane M. Kovacs A. Laurent P. Lindblad K. Gustavsson I. (1999). Simultaneous detection of X- and Y-bearing bull spermatozoa by double colour fluorescence in situ hybridization. *Mol. Reprod. Dev.* 53 407–412. 10.1002/(sici)1098-2795(199908)53:4<407::aid-mrd6>3.0.co;2-o 10398416

[B58] Hassold T. Abruzzo M. Adkins K. Griffin D. Merrill M. Millie E. (1996). Human aneuploidy: incidence, origin, and etiology. *Environ. Mol. Mutagen.* 28 167–175. 10.1002/(sici)1098-2280(1996)28:3<167::aid-em2>3.3.co;2-v 8908177

[B59] Hassold T. Hunt P. (2001). To err (meiotically) is human: the genesis of human aneuploidy. *Nat. Rev. Genet.* 2 280–291. 10.1038/35066065 11283700

[B60] Hendricks K. E. Martins L. Hansen P. J. (2009). Consequences for the bovine embryo of being derived from a spermatozoon subjected to post-ejaculatory aging and heat shock: development to the blastocyst stage and sex ratio. *J. Reprod. Dev.* 55 69–74. 10.1262/jrd.20097 18957823

[B61] Hendriksen P. J. (1999). Do X and Y spermatozoa differ in proteins? *Theriogenology* 52 1295–1307. 10.1016/s0093-691x(99)00218-6 10735077

[B62] Hendriksen P. J. Tieman M. Van Der Lende T. Johnson L. A. (1993). Binding of anti-H-Y monoclonal antibodies to separated X and Y chromosome-bearing porcine and bovine sperm. *Mol. Reprod. Dev.* 35 189–196. 10.1002/mrd.1080350213 8318224

[B63] Hendriksen P. J. Welch G. R. Grootegoed J. A. Van Der Lende T. Johnson L. A. (1996). Comparison of detergent-solubilized membrane and soluble proteins from flow cytometrically sorted X- and Y-chromosome bearing porcine spermatozoa by high resolution 2-D electrophoresis. *Mol. Reprod. Dev.* 45 342–350. 10.1002/(sici)1098-2795(199611)45:3<342::aid-mrd11>3.3.co;2-s 8916045

[B64] Hoegerman S. F. Pang M. G. Kearns W. G. (1995). Sex chromosome abnormalities after intracytoplasmic sperm injection. *Lancet* 346:1095 10.1016/s0140-6736(95)91768-3 7564797

[B65] Hoffmann D. S. Killian G. J. (1981). Isolation of epithelial cells from the corpus epididymidis and analysis for glycerylphosphorylcholine, sialic acid, and protein. *J. Exp. Zool.* 217 93–102. 10.1002/jez.1402170110 7264580

[B66] Holt W. V. Fazeli A. (2010). The oviduct as a complex mediator of mammalian sperm function and selection. *Mol. Reprod. Dev.* 77 934–943. 10.1002/mrd.21234 20886635

[B67] Hoppe P. C. Koo G. C. (1984). Reacting mouse sperm with monoclonal H-Y antibodies does not influence sex ratio of eggs fertilized in vitro. *J. Reprod. Immunol.* 6 1–9. 10.1016/0165-0378(84)90036-6 6694157

[B68] Hossain A. M. Barik S. Kulkarni P. M. (2001). Lack of significant morphological differences between human X and Y spermatozoa and their precursor cells (spermatids) exposed to different prehybridization treatments. *J. Androl.* 22 119–123. 11191075

[B69] Hu Y. C. Namekawa S. H. (2015). Functional significance of the sex chromosomes during spermatogenesis. *Reproduction* 149 R265–R277. 10.1530/REP-14-0613 25948089 PMC4510947

[B70] Iizuka R. Kaneko S. Aoki R. Kobayashi T. (1987). Sexing of human sperm by discontinuous Percoll density gradient and its clinical application. *Hum. Reprod.* 2 573–575. 10.1093/oxfordjournals.humrep.a136591 3680486

[B71] Ikeda M. Tamura M. Yamashita J. Suzuki C. Tomita T. (2005). Repeated in utero and lactational 2,3,7,8-tetrachlorodibenzo-p-dioxin exposure affects male gonads in offspring, leading to sex ratio changes in F2 progeny. *Toxicol. Appl. Pharmacol.* 206 351–355. 10.1016/j.taap.2004.11.019 16039946

[B72] In’t Veld P. Brandenburg H. Verhoeff A. Dhont M. Los F. (1995). Sex chromosomal abnormalities and intracytoplasmic sperm injection. *Lancet* 346:773 10.1016/s0140-6736(95)91531-1 7658889

[B73] Irving J. Bittles A. Peverall J. Murch A. Matson P. (1999). The ratio of X- and Y-bearing sperm in ejaculates of men with three or more children of the same sex. *J. Assist. Reprod. Genet.* 16 492–494. 10530404 10.1023/A:1020555101059 PMC3455630

[B74] Ishihara K. Warita K. Tanida T. Sugawara T. Kitagawa H. Hoshi N. (2007). Does paternal exposure to 2,3,7,8-tetrachlorodibenzo-p-dioxin (TCDD) affect the sex ratio of offspring? *J. Vet. Med. Sci.* 69 347–352. 10.1292/jvms.69.347 17485921

[B75] Iwata H. Shiono H. Kon Y. Matsubara K. Kimura K. Kuwayama T. (2008). Effects of modification of in vitro fertilization techniques on the sex ratio of the resultant bovine embryos. *Anim. Reprod. Sci.* 105 234–244. 10.1016/j.anireprosci.2007.03.006 17391877

[B76] James W. H. Rostron J. (1985). Parental age, parity and sex ratio in births in England and Wales, 1968-77. *J. Biosoc. Sci.* 17 47–56. 10.1017/s0021932000015467 3972856

[B77] Jasin M. Zalamea P. (1992). Analysis of *Escherichia coli* beta-galactosidase expression in transgenic mice by flow cytometry of sperm. *Proc. Natl. Acad. Sci. U.S.A.* 89 10681–10685. 10.1073/pnas.89.22.10681 1438265 PMC50405

[B78] Jeulin C. Wiels J. Casanova M. Fellous M. (1982). “Relationship between Y-chromosome and H-Y antigen expression in human spermatozoa,” in *The Sperm Cell*, ed. André J. (Dordrecht: Springer).

[B79] Johnson L. (1994). A new approach to study the architectural arrangement of spermatogenic stages revealed little evidence of a partial wave along the length of human seminiferous tubules. *J. Androl.* 15 435–441. 7860423

[B80] Johnson L. A. (2000). Sexing mammalian sperm for production of offspring: the state-of-the-art. *Anim. Reprod. Sci.* 6 93–107. 10.1016/s0378-4320(00)00088-9 10844187

[B81] Johnson L. A. Flook J. P. Hawk H. W. (1989). Sex preselection in rabbits: live births from X and Y sperm separated by DNA and cell sorting. *Biol. Reprod.* 41 199–203. 10.1095/biolreprod41.2.199 2804212

[B82] Johnson L. A. Welch G. R. Keyvanfar K. Dorfmann A. Fugger E. F. Schulman J. D. (1993). Gender preselection in humans? Flow cytometric separation of X and Y spermatozoa for the prevention of X-linked diseases. *Hum. Reprod.* 8 1733–1739. 10.1093/oxfordjournals.humrep.a137925 8300839

[B83] Jurewicz J. Radwan M. Sobala W. Radwan P. Jakubowski L. Hawula W. (2014). Lifestyle factors and sperm aneuploidy. *Reprod. Biol.* 14 190–199. 10.1016/j.repbio.2014.02.002 25152516

[B84] Kaneko S. Iizuka R. Oshio S. Nakajima H. Oshio S. Mohri H. (1993). Separation of human X- and Y-bearing sperm using free-flow electrophoresis. *Proc. Jpn. Acad. Ser. B* 59 276–279. 10.2183/pjab.59.276

[B85] Kaneko S. Oshio S. Kobayashi T. Iizuka R. Mohri H. (1984). Human X- and Y-bearing sperm differ in cell surface sialic acid content. *Biochem. Biophys. Res. Commun.* 124 950–955. 10.1016/0006-291x(84)91050-7 6542364

[B86] Kirley T. L. (1990). Inactivation of (Na^+^,K^+^)-ATPase by beta-mercaptoethanol. Differential sensitivity to reduction of the three beta subunit disulfide bonds. *J. Biol. Chem.* 265 4227–4232. 2155215

[B87] Krco C. J. Goldberg E. H. (1976). H-Y male antigen: detection on eight-cell mouse embryos. *Science* 193 1134–1135. 10.1126/science.959826 959826

[B88] Kruger A. N. Brogley M. A. Huizinga J. L. Kidd J. M. de Rooij D. G. Hu Y. C. (2019). A neofunctionalized X-linked ampliconic gene family is essential for male fertility and equal sex ratio in mice. *Curr. Biol.* 29 3699–3706.e5. 10.1016/j.cub.2019.08.057 31630956 PMC7012382

[B89] Kwon W. S. Rahman M. S. Lee J. S. Kim J. Yoon S. J. Park Y. J. (2014a). A comprehensive proteomic approach to identifying capacitation related proteins in boar spermatozoa. *BMC Genomics* 15:897. 10.1186/1471-2164-15-897 25315394 PMC4287242

[B90] Kwon W. S. Rahman M. S. Pang M. G. (2014b). Diagnosis and prognosis of male infertility in mammal: the focusing of tyrosine phosphorylation and phosphotyrosine proteins. *J. Proteome Res.* 13 4505–4517. 10.1021/pr500524p 25223855

[B91] Kwon W. S. Rahman M. S. Lee J. S. Yoon S. J. Park Y. J. Pang M. G. (2015). Discovery of predictive biomarkers for litter size in boar spermatozoa. *Mol. Cell. Proteomics* 14 1230–1240. 10.1074/mcp.M114.045369 25693803 PMC4424395

[B92] Landrum B. Shettles L. B. (1960). Nuclear structure of human spermatozoa. *Nature* 188 916–918. 10.1038/188916a0 13724865

[B93] Lankenau S. Corces V. G. Lankenau D. H. (1994). The Drosophila micropia retrotransposon encodes a testis-specific antisense RNA complementary to reverse transcriptase. *Mol. Cell. Biol.* 14 1764–1775. 10.1128/mcb.14.3.1764 7509447 PMC358534

[B94] Leblond C. P. Clermont Y. (1952). Definition of the stages of the cycle of the seminiferous epithelium in the rat. *Ann. N. Y. Acad. Sci.* 55 548–573. 10.1111/j.1749-6632.1952.tb26576.x 13139144

[B95] Lechniak D. Strabel T. Bousquet D. King A. W. (2003). Sperm pre-incubation prior to insemination affects the sex ratio of bovine embryos produced in vitro. *Reprod. Domest. Anim.* 38 224–227. 10.1046/j.1439-0531.2003.00410.x 12753558

[B96] Lobel S. M. Pomponio R. J. Mutter G. L. (1993). The sex ratio of normal and manipulated human sperm quantitated by the polymerase chain reaction. *Fertil. Steril.* 59 387–392. 10.1016/s0015-0282(16)55682-9 8425636

[B97] Malhotra P. Kumari S. Kumar R. Varma S. (2004). Prevalence of anemia in adult rural population of north India. *J. Assoc. Physicians India* 52 18–20. 15633712

[B98] Martin R. H. Balkan W. Burns K. Rademaker A. W. Lin C. C. Rudd N. L. (1983). The chromosome constitution of 1000 human spermatozoa. *Hum. Genet.* 63 305–309. 10.1007/bf00274750 6683243

[B99] Martin R. H. Rademaker A. W. Leonard N. J. (1995a). Analysis of chromosomal abnormalities in human sperm after chemotherapy by karyotyping and fluorescence in situ hybridization (FISH). *Cancer Genet. Cytogenet.* 80 29–32. 10.1016/0165-4608(94)00162-5 7535187

[B100] Martin R. H. Spriggs E. Ko E. Rademaker A. W. (1995b). The relationship between paternal age, sex ratios, and aneuploidy frequencies in human sperm, as assessed by multicolor FISH. *Am. J. Hum. Genet.* 57 1395–1399. 8533769 PMC1801415

[B101] Martin R. H. Spriggs E. Rademaker A. W. (1996). Multicolor fluorescence in situ hybridization analysis of aneuploidy and diploidy frequencies in 225,846 sperm from 10 normal men. *Biol. Reprod.* 54 394–398. 10.1095/biolreprod54.2.394 8788191

[B102] Mcauliffe M. E. Williams P. L. Korrick S. A. Altshul L. M. Perry M. J. (2012). Environmental exposure to polychlorinated biphenyls and p,p’-DDE and sperm sex-chromosome disomy. *Environ. Health Perspect.* 120 535–540. 10.1289/ehp.1104017 22189045 PMC3339457

[B103] Mcdonald E. Watterson A. Tyler A. N. Mcarthur J. Scott E. M. (2014). Multi-factorial influences on sex ratio: a spatio-temporal investigation of endocrine disruptor pollution and neighborhood stress. *Int. J. Occup. Environ. Health* 20 235–246. 10.1179/2049396714Y.0000000073 25000111 PMC4070448

[B104] Meng L. Feldman L. (2010). A rapid TRIzol-based two-step method for DNA-free RNA extraction from Arabidopsis siliques and dry seeds. *Biotechnol. J.* 5 183–186. 10.1002/biot.200900211 20108272

[B105] Mocarelli P. Gerthoux P. M. Ferrari E. Patterson D. G. Jr. Kieszak S. M. Brambilla P. (2000). Paternal concentrations of dioxin and sex ratio of offspring. *Lancet* 355 1858–1863. 10.1016/s0140-6736(00)02290-x 10866441

[B106] Muehleis P. M. Long S. Y. (1976). The effects of altering the pH of seminal fluid on the sex ratio of rabbit offspring. *Fertil. Steril.* 27 1438–1445. 10.1016/s0015-0282(16)42261-2 12027

[B107] Oakberg E. F. (1956). Duration of spermatogenesis in the mouse and timing of stages of the cycle of the seminiferous epithelium. *Am. J. Anat.* 99 507–516. 10.1002/aja.1000990307 13402729

[B108] Ohno S. Wachtel S. S. (1978). On the selective elimination of Y-bearing sperm. *Immunogenetics* 7 13–16. 10.1007/BF01843982 21302051

[B109] Oyeyipo I. P. van der Linde M. du Plessis S. S. (2017). Environmental exposure of sperm sex-chromosomes: a gender selection technique. *Toxicol. Res.* 33 315–323. 10.5487/TR.2017.33.4.315 29071016 PMC5654200

[B110] Panas M. Karadima G. Vassos E. Kalfakis N. Kladi A. Christodoulou K. (2011). Huntington’s disease in Greece: the experience of 14 years. *Clin. Genet.* 80 586–590. 10.1111/j.1399-0004.2010.01603.x 21166788

[B111] Pang M. G. Hoegerman S. F. Cuticchia A. J. Moon S. Y. Doncel G. F. Acosta A. A. (1999). Detection of aneuploidy for chromosomes 4, 6, 7, 8, 9, 10, 11, 12, 13, 17, 18, 21, X and Y by fluorescence in-situ hybridization in spermatozoa from nine patients with oligoasthenoteratozoospermia undergoing intracytoplasmic sperm injection. *Hum. Reprod.* 14 1266–1273. 10.1093/humrep/14.5.1266 10325276

[B112] Pang M. G. Kim Y. J. Lee S. H. Kim C. K. (2005). The high incidence of meiotic errors increases with decreased sperm count in severe male factor infertilities. *Hum. Reprod.* 20 1688–1694. 10.1093/humrep/deh817 15734753

[B113] Pang M. G. You Y. A. Park Y. J. Oh S. A. Kim D. S. Kim Y. J. (2010). Numerical chromosome abnormalities are associated with sperm tail swelling patterns. *Fertil. Steril.* 94 1012–1020. 10.1016/j.fertnstert.2009.04.043 19505688

[B114] Park Y. J. Kim J. You Y. A. Pang M. G. (2013). Proteomic revolution to improve tools for evaluating male fertility in animals. *J. Proteome Res.* 12 4738–4747. 10.1021/pr400639x 24016215

[B115] Penfold L. M. Holt C. Holt W. V. Welch G. R. Cran D. G. Johnson L. A. (1998). Comparative motility of X and Y chromosome-bearing bovine sperm separated on the basis of DNA content by flow sorting. *Mol. Reprod. Dev.* 50 323–327. 10.1002/(sici)1098-2795(199807)50:3<323::aid-mrd8>3.0.co;2-l 9621308

[B116] Pepin J. Mpia B. Iloasebe M. (2002). *Trypanosoma brucei gambiense* African trypanosomiasis: differences between men and women in severity of disease and response to treatment. *Trans. R. Soc. Trop. Med. Hyg.* 96 421–426. 10.1016/s0035-9203(02)90380-9 12497980

[B117] Perez-Crespo M. Pintado B. Gutierrez-Adan A. (2008). Scrotal heat stress effects on sperm viability, sperm DNA integrity, and the offspring sex ratio in mice. *Mol. Reprod. Dev.* 75 40–47. 10.1002/mrd.20759 17474098

[B118] Perry M. J. (2008). Effects of environmental and occupational pesticide exposure on human sperm: a systematic review. *Hum. Reprod. Update* 14 233–242. 10.1093/humupd/dmm039 18281240

[B119] Pfeffer J. Pang M. G. Hoegerman S. F. Osgood C. J. Stacey M. W. Mayer J. (1999). Aneuploidy frequencies in semen fractions from ten oligoasthenoteratozoospermic patients donating sperm for intracytoplasmic sperm injection. *Fertil. Steril.* 72 472–478. 10.1016/s0015-0282(99)00279-4 10519619

[B120] Pratt N. C. Huck U. W. Lisk R. D. (1987). Offspring sex ratio in hamsters is correlated with vaginal pH at certain times of mating. *Behav. Neural Biol.* 48 310–316. 10.1016/s0163-1047(87)90864-8 3675523

[B121] Quinlivan W. L. Preciado K. Long T. L. Sullivan H. (1982). Separation of human X and Y spermatozoa by albumin gradients and Sephadex chromatography. *Fertil. Steril.* 37 104–107. 10.1016/s0015-0282(16)45986-8 6174374

[B122] Quinlivan W. L. Sullivan H. (1974). The ratios and separation of X and Y spermatozoa in human semen. *Fertil. Steril.* 25 315–318. 10.1016/s0015-0282(16)40330-4 4132058

[B123] Rahman M. S. Kang K. H. Arifuzzaman S. Pang W. K. Ryu D. Y. Song W. H. (2019). Effect of antioxidants on BPA-induced stress on sperm function in a mouse model. *Sci. Rep.* 9:10584. 10.1038/s41598-019-47158-9 31332285 PMC6646364

[B124] Rahman M. S. Kwon W. S. Karmakar P. C. Yoon S. J. Ryu B. Y. Pang M. G. (2017a). Gestational exposure to bisphenol A affects the function and proteome profile of F1 spermatozoa in adult mice. *Environ. Health Perspect.* 125 238–245. 10.1289/EHP378 27384531 PMC5289913

[B125] Rahman M. S. Kwon W. S. Pang M. G. (2017b). Prediction of male fertility using capacitation-associated proteins in spermatozoa. *Mol. Reprod. Dev.* 84 749–759. 10.1002/mrd.22810 28390187

[B126] Rahman M. S. Kwon W. S. Lee J. S. Yoon S. J. Ryu B. Y. Pang M. G. (2015). Bisphenol-A affects male fertility via fertility-related proteins in spermatozoa. *Sci. Rep.* 5:9169. 10.1038/srep09169 25772901 PMC4360475

[B127] Rahman M. S. Kwon W. S. Ryu D. Y. Khatun A. Karmakar P. C. Ryu B. Y. (2018). Functional and proteomic alterations of F1 capacitated spermatozoa of adult mice following gestational exposure to bisphenol A. *J. Proteome Res.* 17 524–535. 10.1021/acs.jproteome.7b00668 29198108

[B128] Rahman M. S. Kwon W. S. Yoon S. J. Park Y. J. Ryu B. Y. Pang M. G. (2016). A novel approach to assessing bisphenol-A hazards using an *in vitro* model system. *BMC Genomics* 17:577. 10.1186/s12864-016-2979-5 27507061 PMC4977886

[B129] Rahman M. S. Lee J. S. Kwon W. S. Pang M. G. (2013). Sperm proteomics: road to male fertility and contraception. *Int. J. Endocrinol.* 2013:360986. 10.1155/2013/360986 24363670 PMC3864079

[B130] Rathje C. C. Johnson E. E. P. Drage D. Patinioti C. Silvestri G. Affara N. A. (2019). Differential sperm motility mediates the sex ratio drive shaping mouse sex chromosome evolution. *Curr. Biol.* 29 3692–3698.e4. 10.1016/j.cub.2019.09.031 31630954 PMC6839398

[B131] Recio R. Robbins W. A. Borja-Aburto V. Moran-Martinez J. Froines J. R. Hernandez R. M. (2001). Organophosphorous pesticide exposure increases the frequency of sperm sex null aneuploidy. *Environ. Health Perspect.* 109 1237–1240. 10.1289/ehp.011091237 11748030 PMC1240505

[B132] Ross A. Robinson J. A. Evans H. J. (1975). Failure to confirm separation of X- and Y-bearing human sperm using BSA gradients. *Nature* 253 354–355. 10.1038/253354a0 46109

[B133] Rubio C. Gil-Salom M. Simon C. Vidal F. Rodrigo L. Minguez Y. (2001). Incidence of sperm chromosomal abnormalities in a risk population: relationship with sperm quality and ICSI outcome. *Hum. Reprod.* 16 2084–2092. 10.1093/humrep/16.10.2084 11574496

[B134] Ruder A. (1985). Paternal-age and birth-order effect on the human secondary sex ratio. *Am. J. Hum. Genet.* 37 362–372. 3985011 PMC1684568

[B135] Ryan J. J. Amirova Z. Carrier G. (2002). Sex ratios of children of Russian pesticide producers exposed to dioxin. *Environ. Health Perspect.* 110 A699–A701. 12417498 10.1289/ehp.021100699 PMC1241090

[B136] Salicioni A. M. Platt M. D. Wertheimer E. V. Arcelay E. Allaire A. Sosnik J. (2007). Signalling pathways involved in sperm capacitation. *Soc. Reprod. Fertil. Suppl.* 65 245–259. 17644966

[B137] Samura O. Miharu N. He H. Okamoto E. Ohama K. (1997). Assessment of sex chromosome ratio and aneuploidy rate in motile spermatozoa selected by three different methods. *Hum. Reprod.* 12 2437–2442. 10.1093/humrep/12.11.2437 9436680

[B138] Sarkar S. Jolly D. J. Friedmann T. Jones O. W. (1984). Swimming behavior of X and Y human sperm. *Differentiation* 27 120–125. 10.1111/j.1432-0436.1984.tb01416.x 6541168

[B139] Schmidt W. Jenderny J. Hecher K. Hackeloer B. J. Kerber S. Kochhan L. (2000). Detection of aneuploidy in chromosomes X, Y, 13, 18 and 21 by QF-PCR in 662 selected pregnancies at risk. *Mol. Hum. Reprod.* 6 855–860. 10.1093/molehr/6.9.855 10956559

[B140] Scott C. De Souza F. F. Aristizabal V. H. V. Hethrington L. Krisp C. Molloy M. (2018). Proteomic profile of sex-sorted bull sperm evaluated by SWATH-MS analysis. *Anim. Reprod. Sci.* 198 121–128. 10.1016/j.anireprosci.2018.09.010 30274742

[B141] Shannon M. Handel M. A. (1993). Expression of the Hprt gene during spermatogenesis: implications for sex-chromosome inactivation. *Biol. Reprod.* 49 770–778. 10.1095/biolreprod49.4.770 8218641

[B142] Shettles L. B. (1960). Nuclear morphology of human spermatozoa. *Nature* 186 648–649. 10.1038/186648a0 14445926

[B143] Shettles L. B. (1961). Human spermatozoa shape in relation to sex ratios. *Fertil. Steril.* 12 502–508. 10.1016/s0015-0282(16)34321-7 13911772

[B144] Shettles L. B. (1970). Factors influencing sex ratios. *Int. J. Gynecol. Obstet.* 8 643–647. 10.1002/j.1879-3479.1970.tb00029.x

[B145] Siegel R. L. Miller K. D. Jemal A. (2015). Cancer statistics, 2015. *CA Cancer J. Clin.* 65 5–29. 10.3322/caac.21254 25559415

[B146] Sills E. S. Kirman I. Colombero L. T. Hariprashad J. Rosenwaks Z. Palermo G. D. (1998). H-Y antigen expression patterns in human X- and Y-chromosome-bearing spermatozoa. *Am. J. Reprod. Immunol.* 40 43–47. 10.1111/j.1600-0897.1998.tb00387.x 9689360

[B147] Silvers W. K. Wachtel S. S. (1977). H-Y antigen: behavior and function. *Science* 195 956–960. 10.1126/science.320662 320662

[B148] Smith B. E. Braun R. E. (2012). Germ cell migration across Sertoli cell tight junctions. *Science* 338 798–802. 10.1126/science.1219969 22997133 PMC3694388

[B149] Smith J. L. Garry V. F. Rademaker A. W. Martin R. H. (2004). Human sperm aneuploidy after exposure to pesticides. *Mol. Reprod. Dev.* 67 353–359. 10.1002/mrd.20022 14735496

[B150] Song W. H. Mohamed E. A. Pang W. K. Kang K. H. Ryu D. Y. Rahman M. S. (2018). Effect of endocrine disruptors on the ratio of X and Y chromosome-bearing live spermatozoa. *Reprod. Toxicol.* 82 10–17. 10.1016/j.reprotox.2018.09.002 30219569

[B151] Spriggs E. L. Rademaker A. W. Martin R. H. (1996). Aneuploidy in human sperm: the use of multicolor FISH to test various theories of nondisjunction. *Am. J. Hum. Genet.* 58 356–362. 8571962 PMC1914531

[B152] Szyda J. Simianer H. Lien S. (2000). Sex ratio distortion in bovine sperm correlates to recombination in the pseudoautosomal region. *Genet. Res.* 75 53–59. 10.1017/s0016672399004085 10740921

[B153] Templado C. Bosch M. Benet J. (2005). Frequency and distribution of chromosome abnormalities in human spermatozoa. *Cytogenet. Genome Res.* 111 199–205. 10.1159/000086890 16192695

[B154] Terrell M. L. Hartnett K. P. Marcus M. (2011). Can environmental or occupational hazards alter the sex ratio at birth? A systematic review. *Emerg. Health Threats J.* 4:7109. 10.3402/ehtj.v4i0.7109 24149027 PMC3168220

[B155] Tesarik J. Mendoza C. (1996). Genomic imprinting abnormalities: a new potential risk of assisted reproduction. *Mol. Hum. Reprod.* 2 295–298. 10.1093/molehr/2.5.295 9238695

[B156] Ueda K. Yanagimachi R. (1987). Sperm chromosome analysis as a new system to test human X- and Y-sperm separation. *Gamete Res.* 17 221–228. 10.1002/mrd.1120170305 3507349

[B157] Umehara T. Tsujita N. Shimada M. (2019). Activation of Toll-like receptor 7/8 encoded by the X chromosome alters sperm motility and provides a novel simple technology for sexing sperm. *PLoS Biol.* 17:e3000398. 10.1371/journal.pbio.3000398 31408454 PMC6691984

[B158] Utsumi K. Hayashi M. Takakura R. Utaka K. Iritani A. (1993). Embryo sex selection by a rat male-specific antibody and the cytogenetic and developmental confirmation in cattle embryos. *Mol. Reprod. Dev.* 34 25–32. 10.1002/mrd.1080340105 8418813

[B159] Van Dyk Q. Mahony M. C. Hodgen G. D. (2001). Differential binding of X- and Y-chromosome-bearing human spermatozoa to zona pellucida in vitro. *Andrologia* 33 199–205. 10.1046/j.1439-0272.2001.00427.x 11472331

[B160] Van Kooij R. J. Van Oost B. A. (1992). Determination of sex ratio of spermatozoa with a deoxyribonucleic acid-probe and quinacrine staining: a comparison. *Fertil. Steril.* 58 384–386. 10.1016/s0015-0282(16)55226-1 1378794

[B161] Van Larebeke N. A. Sasco A. J. Brophy J. T. Keith M. M. Gilbertson M. Watterson A. (2008). Sex ratio changes as sentinel health events of endocrine disruption. *Int. J. Occup. Environ. Health* 14 138–143. 10.1179/oeh.2008.14.2.138 18507291

[B162] Van Munster E. B. Stap J. Hoebe R. A. Te Meerman G. J. Aten J. A. (1999). Difference in volume of X- and Y-chromosome-bearing bovine sperm heads matches difference in DNA content. *Cytometry* 35 125–128. 10.1002/(sici)1097-0320(19990201)35:2<125::aid-cyto3>3.0.co;2-h 10554167

[B163] Van Opstal D. Los F. J. Ramlakhan S. Van Hemel J. O. Van Den Ouweland A. M. Brandenburg H. (1997). Determination of the parent of origin in nine cases of prenatally detected chromosome aberrations found after intracytoplasmic sperm injection. *Hum. Reprod.* 12 682–686. 10.1093/humrep/12.4.682 9159424

[B164] Vina J. Lloret A. (2010). Why women have more Alzheimer’s disease than men: gender and mitochondrial toxicity of amyloid-beta peptide. *J. Alzheimers Dis.* 20(Suppl. 2), S527–S533. 10.3233/JAD-2010-100501 20442496

[B165] Visconti P. E. (2009). Understanding the molecular basis of sperm capacitation through kinase design. *Proc. Natl. Acad. Sci. U.S.A.* 106 667–668. 10.1073/pnas.0811895106 19144927 PMC2630107

[B166] Visconti P. E. (2012). Sperm bioenergetics in a nutshell. *Biol. Reprod.* 87:72. 10.1095/biolreprod.112.104109 PMC3464909 22914312

[B167] Wang H. X. Flaherty S. P. Swann N. J. Matthews C. D. (1994). Discontinuous Percoll gradients enrich X-bearing human spermatozoa: a study using double-label fluorescence in-situ hybridization. *Hum. Reprod.* 9 1265–1270. 10.1093/oxfordjournals.humrep.a138692 7962431

[B168] Ward W. S. Coffey D. S. (1991). DNA packaging and organization in mammalian spermatozoa: comparison with somatic cells. *Biol. Reprod.* 44 569–574. 10.1095/biolreprod44.4.569 2043729

[B169] Williams B. J. Ballenger C. A. Malter H. E. Bishop F. Tucker M. Zwingman T. A. (1993). Non-disjunction in human sperm: results of fluorescence in situ hybridization studies using two and three probes. *Hum. Mol. Genet.* 2 1929–1936. 10.1093/hmg/2.11.1929 8281157

[B170] Windsor D. P. Evans G. White I. G. (1993). Sex predetermination by separation of X and Y chromosome-bearing sperm: a review. *Reprod. Fertil. Dev.* 5 155–171. 8265800 10.1071/rd9930155

[B171] Woldrich J. M. Mallin K. Ritchey J. Carroll P. R. Kane C. J. (2008). Sex differences in renal cell cancer presentation and survival: an analysis of the National Cancer Database, 1993-2004. *J. Urol.* 179 1709–1713; discussion 1713. 10.1016/j.juro.2008.01.024 18343430

[B172] Wu E. M. Wong L. L. Hernandez B. Y. Ji J. F. Jia W. Kwee S. A. (2018). Gender differences in hepatocellular cancer: disparities in nonalcoholic fatty liver disease/steatohepatitis and liver transplantation. *Hepatoma Res.* 4:66. 10.20517/2394-5079.2018.87 30687780 PMC6347119

[B173] Xia Y. Cheng S. Bian Q. Xu L. Collins M. D. Chang H. C. (2005). Genotoxic effects on spermatozoa of carbaryl-exposed workers. *Toxicol. Sci.* 85 615–623. 10.1093/toxsci/kfi066 15615886

[B174] Yan J. Feng H. L. Chen Z. J. Hu J. Gao X. Qin Y. (2006). Influence of swim-up time on the ratio of X- and Y-bearing spermatozoa. *Eur. J. Obstet. Gynecol. Reprod. Biol.* 129 150–154. 10.1016/j.ejogrb.2006.02.020 16678330

[B175] You Y. A. Kwon W. S. Saidur Rahman M. Park Y. J. Kim Y. J. Pang M. G. (2017). Sex chromosome-dependent differential viability of human spermatozoa during prolonged incubation. *Hum. Reprod.* 32 1183–1191. 10.1093/humrep/dex080 28430968

[B176] You Y. A. Mohamed E. A. Rahman M. S. Kwon W. S. Song W. H. Ryu B. Y. (2018). 2,3,7,8-Tetrachlorodibenzo-p-dioxin can alter the sex ratio of embryos with decreased viability of Y spermatozoa in mice. *Reprod. Toxicol.* 77 130–136. 10.1016/j.reprotox.2018.02.011 29505796

[B177] Zavaczki Z. Celik-Ozenci C. Ovari L. Jakab A. Sati G. L. Ward D. C. (2006). Dimensional assessment of X-bearing and Y-bearing haploid and disomic human sperm with the use of fluorescence in situ hybridization and objective morphometry. *Fertil. Steril.* 85 121–127. 10.1016/j.fertnstert.2005.07.1295 16412741

[B178] Zhu J. Barratt C. L. Lippes J. Pacey A. A. Lenton E. A. Cooke I. D. (1994). Human oviductal fluid prolongs sperm survival. *Fertil. Steril.* 61 360–366. 10.1016/s0015-0282(16)56532-7 8299797

